# A technical review of canonical correlation analysis for neuroscience applications

**DOI:** 10.1002/hbm.25090

**Published:** 2020-06-27

**Authors:** Xiaowei Zhuang, Zhengshi Yang, Dietmar Cordes

**Affiliations:** ^1^ Cleveland Clinic Lou Ruvo Center for Brain Health Las Vegas Nevada USA; ^2^ University of Colorado Boulder Colorado USA; ^3^ Department of Brain Health University of Nevada Las Vegas Nevada USA

**Keywords:** canonical correlation analysis, multivariate analysis, neuroscience

## Abstract

Collecting comprehensive data sets of the same subject has become a standard in neuroscience research and uncovering multivariate relationships among collected data sets have gained significant attentions in recent years. Canonical correlation analysis (CCA) is one of the powerful multivariate tools to jointly investigate relationships among multiple data sets, which can uncover disease or environmental effects in various modalities simultaneously and characterize changes during development, aging, and disease progressions comprehensively. In the past 10 years, despite an increasing number of studies have utilized CCA in multivariate analysis, simple conventional CCA dominates these applications. Multiple CCA‐variant techniques have been proposed to improve the model performance; however, the complicated multivariate formulations and not well‐known capabilities have delayed their wide applications. Therefore, in this study, a comprehensive review of CCA and its variant techniques is provided. Detailed technical formulation with analytical and numerical solutions, current applications in neuroscience research, and advantages and limitations of each CCA‐related technique are discussed. Finally, a general guideline in how to select the most appropriate CCA‐related technique based on the properties of available data sets and particularly targeted neuroscience questions is provided.

## Graphical abstract

Neuroscience applications of canonical correlation analysis (CCA) and its variants are systematically reviewed from a technical perspective. Detailed formulations, analytical and numerical solutions, current applications, and advantages and limitations of CCA and its variants are discussed. A general guideline to select the most appropriate CCA‐related technique is provided.

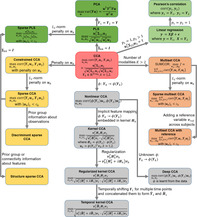

## 1 INTRODUCTION

Recently in neuroscience research, multiple types of data are usually collected from the same individual, including demographics, clinical symptoms, behavioral and neuropsychological measures, genetic information, structural and functional magnetic resonance imaging (fMRI) data, position emission tomography (PET) data, functional near‐infrared spectroscopy (fNIRS) data, and electrophysiological data. Each of these data types, termed modality here, contains multiple measurements and provides a unique view of the subject. These measurements can be the raw data (e.g., neuropsychological tests) or derived information (e.g., brain regional volume and thickness measures derived from T1‐weighted MRI).

Neuroscience research has been focused on uncovering associations between measurements from multiple modalities. Conventionally, a single measurement is selected from each modality, and their one‐to‐one univariate association is analyzed. Multiple correction is then performed to guarantee statistically meaningful results. These univariate associations have illuminated numerous findings in various neurological diseases, such as association between gray‐matter density and Mini Mental State Examination score in Alzheimer's disease (Baxter et al., 2006), correlation between brain network temporal dynamics and Unified Parkinson Disease Rating Scale part III motor scores in Parkinson's disease subjects (Zhuang et al., 2018), and relationship between imaging biomarkers and cognitive performances in fighters with repetitive head trauma (Mishra et al., 2017).

However, the one‐to‐one univariate association overlooks the multivariate joint relationship among multiple measurements between modalities. Furthermore, when dealing with brain imaging data, highly correlated noise further decreases the effectiveness and sensitivity of mass‐univariate voxel‐wise analysis (Cremers, Wager, & Yarkoni, 2017; Zhuang et al., 2017), and different methods of multiple corrections might lead to various statistically meaningful results. Multivariate analysis, alternatively, uncovers the joint covariate patterns among different modalities and avoids multiple correction steps, which would be more appropriate to disentangle joint relationship between modalities and guarantees full utilization of all common information.

Canonical correlation analysis (CCA) is one candidate to uncover these joint multivariate relationships among different modalities. CCA is a statistical method that finds linear combinations of two random variables so that the correlation between the combined variables is maximized (Hotelling, 1936). CCA can identify the source of common statistical variations among multiple modalities, without assuming any particular form of directionality, which suits neuroscience applications. In practice, CCA has been mainly implemented as a substitute for univariate general linear model (GLM) to link different modalities, and therefore, is a major and powerful tool in multimodal data fusion. Multiple CCA variants, including kernel CCA, constrained CCA, deep CCA, and multiset CCA, also have been applied in neuroscience research. However, the complicated multivariate formulations and obscure capabilities remain obstacles for CCA and its variants to being widely applied.

In this study, we review CCA applications in neuroscience research from a technical perspective to improve the understanding of the CCA technique itself and to provide neuroscience researchers with guidlines of proper CCA applications. We briefly discuss studies through December 2019 that have utilized CCA and its variants to uncover the association between multiple modalities. We explain the existing CCA method and its variants for their formulations, properties, relationships to other multivariate techniques, and advantages and limitations in neuroscience applications. We finally provide a flowchart and an experimental example to assist researchers to select the most appropriate CCA technique based on their specific applications.

## 2 INCLUSION/EXCLUSION OF STUDIES

**FIGURE 1:**
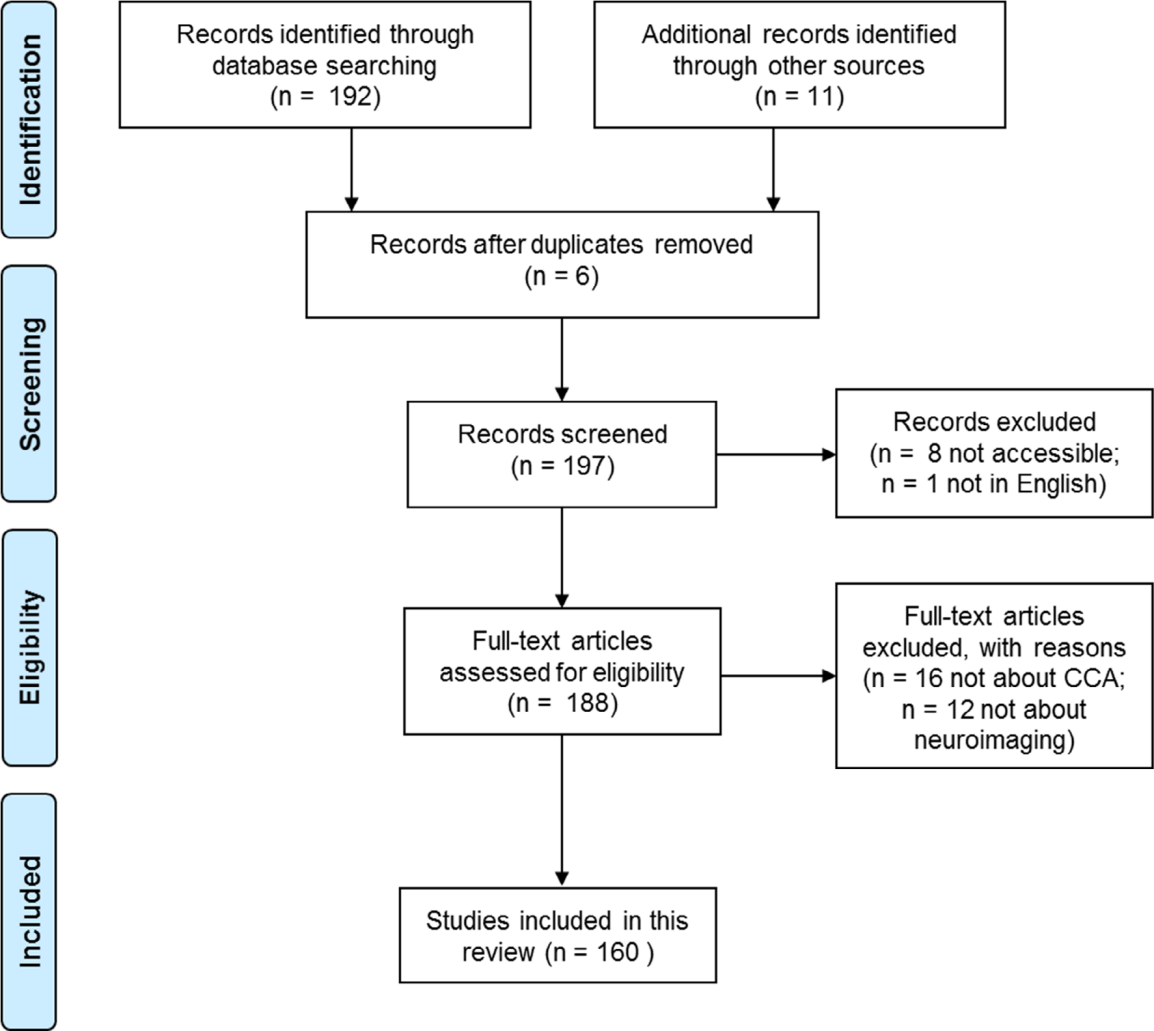
Inclusion and exclusion criteria for this review

Using the PubMed search engine in December 2019, we searched neuroimaging or neuroscience articles using CCA with the following string: (“canonical correlation” analysis) AND (neuroscience OR neuroimaging). This search yielded 192 articles; 11 additional articles were included based on authors' preidentification. We excluded non‐English articles, conference abstracts and duplicated studies, yielding 188 articles assessed for eligibility. We further identified 160 studies that met the following criteria: (a) primarily focused on a CCA or CCA‐variant technique and (b) with an application to neuroimaging or neuroscience modalities. Reasons for exclusion and numbers of articles meeting exclusion criteria at each stage are shown in Figure 1.

**FIGURE 2:**
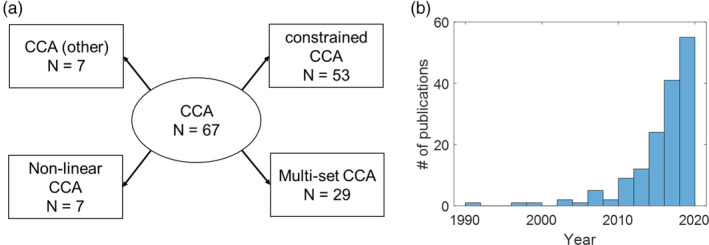
Number of articles summarized by category (a) and year (b)

The remaining articles were full‐text reviewed and divided into five categories based on the applied CCA technique (Figure 2a): CCA (*N* = 67); constrained CCA (*N* = 53); nonlinear CCA (*N* = 7); multiset CCA (*N* = 29); and CCA‐other (*N* = 7). Three articles applied constrained multiset CCA, thus are categorized into both constrained CCA and multiset CCA. Numbers of articles of every year from 1990 to 2019 are plotted in Figure 2 (B).

In the following sections, we present technical details (Section 3) and neuroscience applications for each category (Section 4). In Section 5, we discuss technical differences and summarize advantages and limitations of each CCA‐related technique. We finally provide an experimental example and guidance in Section 6 to researchers who are interested in applying multivariate CCA‐related techniques in their work.

## 3 TECHNICAL DETAILS

**FIGURE 3:**
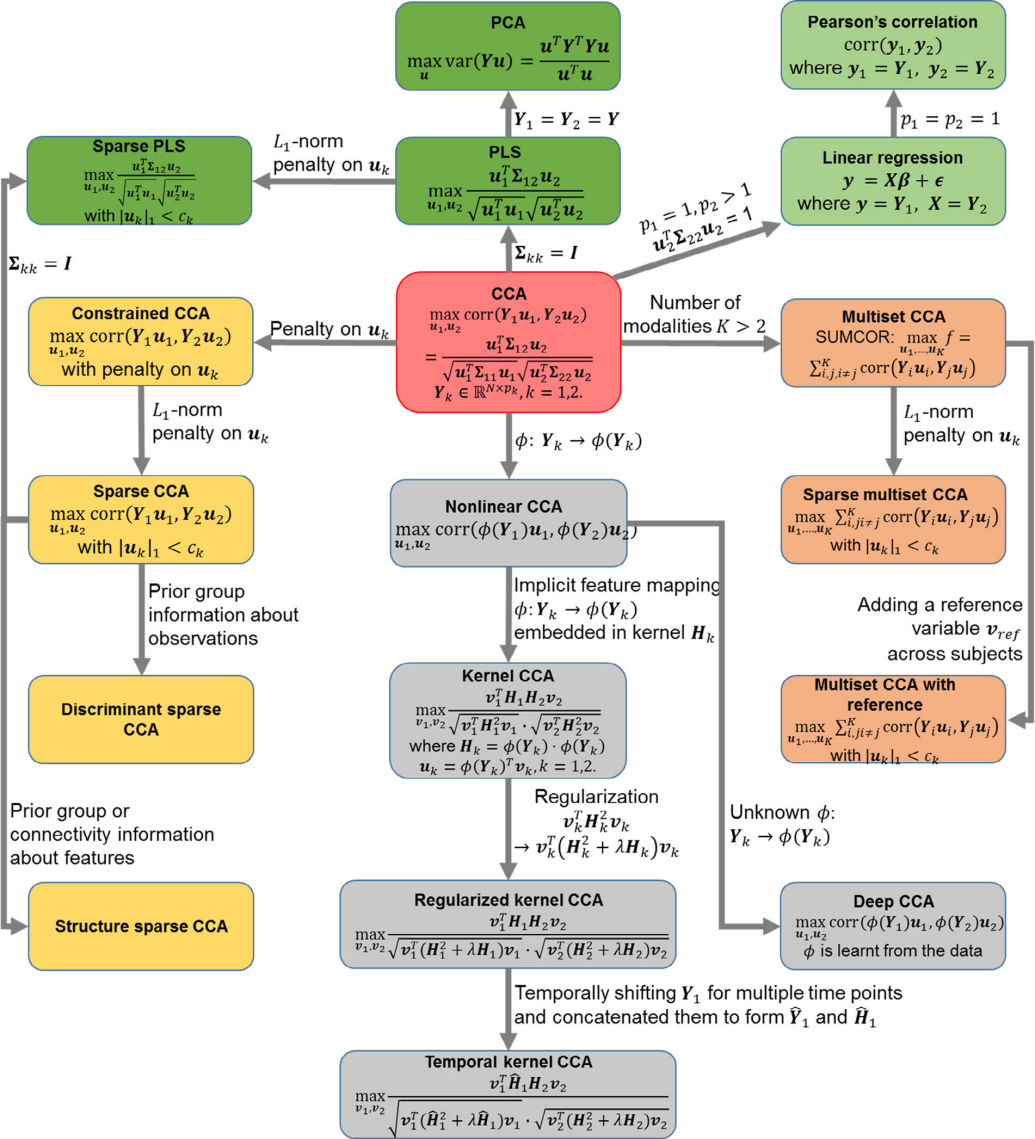
Technical details of CCA and relationship between CCA and its variants. Background color indicates different techniques: red: conventional CCA; gray: nonlinear CCA; yellow: constrained CCA; orange: multiset CCA; green: other techniques related to CCA. CCA, canonical correlation analysis; PCA, principle component analysis; PLS, partial least square

Figure 3 shows the detailed CCA equations (red box) and linkages between CCA and its variants. Constrained CCA (yellow boxes), nonlinear CCA (gray boxes), and multiset CCA (orange boxes) are focused, and linkages between CCA and other univariate (light green boxes) and multivariate (dark green boxes) techniques are also included. Here, we provide basic formulations and solutions of each CCA and its variants. We also discuss how CCA is mathematically linked to its variants and to other multivariate or univariate techniques. Researchers interested in further details can refer to the corresponding references.

### 3.1 Conventional CCA

3.1.1

3.1.1.1

###### Formulations

CCA is designed to maximize the correlation between two latent variables $y_{1}\in R^{p_{1}\times 1}$ and $y_{2}\in R^{p_{2}\times 1}$, which are also being referred to as modalities. Here, we denote $Y_{k}\in R^{N\times p_{k}},k=1,2$ as collected samples of these two variables, where *N* represents the number of observations (samples) and *p*_*k*_, *k* = 1, 2 represent the number of features in each variable. CCA determines the canonical coefficients $u_{1}\in R^{p_{1}\times 1}$ and $u_{2}\in R^{p_{2}\times 1}$ for ***Y***_1_ and ***Y***_2_, respectively, by maximizing the correlation between ***Y***_1_***u***_1_ and ***Y***_2_***u***_2_:

(1) $$\mathrm{CCA}:\max_{u_{1},u_{2}}\rho =\text{corr}\left(Y_{1}u_{1},Y_{2}u_{2}\right)=\frac{u_{1}^{T}{\mathrm{0-k\sum}}_{12}u_{2}}{\sqrt{u_{1}^{T}\sum_{11}u_{1}}\sqrt{u_{2}^{T}\sum_{22}u_{2}}}.$$

In Equation (1), **∑**_11_ and **∑**_22_ are the within‐set covariance matrices and **∑**_12_ is the between‐set covariance matrix. The denominator in Equation (1) is used to normalize within‐set covariance, which guarantees that CCA is invariant to the scaling of coefficients.

###### Solutions

Canonical coefficients ***u***_1_ and ***u***_2_ can be found by setting the partial derivative of the objective function (Equation (1)) with respect to ***u***_1_ and ***u***_2_ to zero, respectively, leading to:

(2) $$\sum_{12}u_{2}={\rho \sum }_{11}u_{1}\;\text{and}\;\sum_{21}u_{1}={\rho \sum }_{22}u_{2}.$$

Equation (2) can be further reduced to a classical eigenvalue problem, if **∑**_*kk*_ is invertible, as follows:

(3) $$\begin{array}{c} \sum_{11}^{-1}\sum_{12}\sum_{22}^{-1}\sum_{21}u_{1}=\rho^{2}u_{1}\\ \sum_{22}^{-1}\sum_{21}\sum_{11}^{-1}\sum_{12}u_{2}=\rho^{2}u_{2} \end{array}.$$

Each pair of canonical coefficients {***u***_1_, ***u***_2_} are the eigenvectors of $\sum_{11}^{-1}\sum_{12}\sum_{22}^{-1}\sum_{21}$ and $\sum_{22}^{-1}\sum_{21}\sum_{11}^{-1}\sum_{12}$, respectively with the same eigenvalue *ρ*^2^. Following Equation (3), up to *M* = min(*p*_1_, *p*_2_) pairs of canonical coefficients can be achieved through singular value decomposition (SVD), and every pair of canonical variables $\left\{Y_{1}u_{1}^{\left(m\right)},Y_{2}u_{2}^{\left(m\right)}\right\},m=1,2,\ldots ,M$, are uncorrelated with another pair of canonical variables. Corresponding *M* canonical correlation values are in descending order as *ρ*^(1)^ > *ρ*^(2)^ > … > *ρ*^(*M*)^.

As we stated above, one requirement for solving the CCA problem (Equation (1)) through this eigenvalue problem (Equation (3)) is that within‐set covariance matrices **∑**_11_ and **∑**_22_ must be invertible. To satisfy this requirement, the number of observations in ***Y***_1_ and ***Y***_2_ should be greater than the number of features, that is, *N* > *p*_*k*_, *k* = 1, 2. Furthermore, since the square of canonical correlation values (*ρ*^2^) are the eigenvalues of matrices $\sum_{11}^{-1}\sum_{12}\sum_{22}^{-1}\sum_{21}$ and $\;\sum_{22}^{-1}\sum_{21}\sum_{11}^{-1}\sum_{12},$ both matrices are required to be positive definite.

###### Statistical inferences

Parametric inferences exist for CCA if both variables strictly follow the Gaussian distribution. The null hypothesis is that no (zero) canonical correlation exists between ***Y***_1_ and ***Y***_2_, that is, *ρ*^(1)^ = *ρ*^(2)^ = … = *ρ*^(*M*)^ = 0. The alternative hypothesis is that at least one canonical correlation value is nonzero. A test statistic based on Wilk's Λ is (Bartlett, 1939):

(4) $$\Lambda =-\left(N-\frac{p_{1}+p_{2}+3}{2}\right)\log\prod_{i=1}^{M}\left(1-\rho^{\left(i\right)}\right),$$

which follows a chi‐square distribution $\chi_{p_{1}\times p_{2}}^{2}$ with degree of freedom of *p*_1_ × *p*_2_. It is also of interest to test if a specific canonical correlation value (*ρ*^(*m*)^, 1 ≤ *m* ≤ *M*) is different from zero. In this case, the test statistic in Equation (4) becomes:

(5) $$\Lambda^{\left(m\right)}=-\left(N-\frac{p_{1}+p_{2}+3}{2}\right)\log\prod_{i=m+1}^{M}\left(1-\rho^{\left(i\right)}\right),$$

which follows $\chi_{\left(p_{1}-m\right)\left(p_{2}-m\right)}^{2}$.

In practice, this parametric inference is not commonly used since it requires variables to strictly follow the Gaussian distribution and is sensitive to outliers (Bartlett, 1939). Instead, permutation‐based nonparametric statistics have been widely used in CCA applications. In general, observations of one variable are randomly shuffled (***Y***_1_ becomes $\hat{Y_{1}}$) while observations of the other variable are kept intact (***Y***_2_ remains). A new set of canonical correlation values are then computed for $\hat{Y_{1}}$ and ***Y***_2_ following Equation (3). This random shuffling is repeated multiple times, and the null distribution of canonical correlation values is generated. Statistical significance (*p*‐values) for the true canonical correlation values are finally obtained from this null distribution.

### 3.2 CCA variants

The conventional CCA (Equation (1)) can be modified for different purposes. Constrained CCA penalizes canonical coefficients ***u***_1_ and ***u***_2_ to satisfy certain requirements and more specifically, to avoid overfitting and unstable results caused by insufficient observations in ***Y***_1_ or ***Y***_2_. Kernel and deep CCA are designed to uncover nonlinear correlations between modalities by projecting the original variables to new nonlinear feature spaces. Multiset CCA is proposed to find multivariate associations among more than two modalities. In this section, we systematically review constrained CCA, nonlinear CCA, multiset CCA, and other special CCA cases.

#### 3.2.1 Constrained CCA

##### Generalized constrained CCA

###### Formulation

Constrained CCA is implemented by adding penalties to coefficients ***u***_*k*_ in Equation (1). Penalties can be either equality constraints or inequality constraints, and based on researcher's own considerations, penalties can be added to either ***u***_1_ or ***u***_2_, or to both ***u***_1_ and ***u***_2_. Therefore, in general, the constrained CCA problem can be formulated in terms of the constrained optimization problem as:

(6) $$\max_{u_{1},u_{2}}\rho =\text{corr}\left(Y_{1}u_{1},Y_{2}u_{2}\right)=\frac{u_{1}^{T}\sum_{12}u_{2}}{\sqrt{u_{1}^{T}\sum_{11}u_{1}}\sqrt{u_{2}^{T}\sum_{22}u_{2}}};\;s.t.\left\{\begin{array}{c} {\mathrm{con}}_{i}\left(u_{1},u_{2}\right)=0,\forall i\in E;\\ {\mathrm{con}}_{j}\left(u_{1},u_{2}\right)>0,\forall j\in \mathrm{InE}; \end{array}\right)$$

where ***E*** represents the set of equality constraints and ***InE*** represents the set of inequality constraints.

###### Solution

Analytical solutions usually do not exist for constrained CCA problems, and solving Equation (6) requires numerical solutions through iterative optimization techniques. Multiple optimization techniques can be applied, such as the Broyden–Fletcher–Goldfarb–Shanno algorithm, augmented‐Lagrangian algorithm, reduced gradient method and sequential quadratic programming. Examples and details of solving constrained CCA problems through above optimization techniques can be found in Yang, Zhuang, et al. (2018) and Zhuang et al. (2017).

##### Special case: *L*_1_‐norm penalty and sparse CCA

###### Formulation

The most commonly implemented penalty in constrained CCA is the *L*_1_‐norm penalty added to either ***u***_1_ or ***u***_2_, and is termed sparse CCA:

(7) $$\text{sparse}\;\mathrm{CCA}:\max_{u_{1},u_{2}}\rho =\text{corr}\left(Y_{1}u_{1},Y_{2}u_{2}\right)=\frac{u_{1}^{T}\sum_{12}u_{2}}{\sqrt{u_{1}^{T}\sum_{11}u_{1}}\sqrt{u_{2}^{T}\sum_{22}u_{2}}};\;s.t.{\left|u_{1}\right|}_{1}<c_{1},{\left|u_{2}\right|}_{1}<c_{2},$$

where |***u***_*i*_|_1_ < *c*_*i*_ are inequality constraints.

The *L*_1_‐norm penalty induces sparsity on canonical coefficients, and therefore sparse CCA can be implemented to high‐dimensional variables. When dealing with high‐dimensional variables, the within‐set covariance matrices **∑**_11_ and **∑**_22_ in Equation (7) are also high‐dimensional matrices, which are memory intensive. In addition, when the number of observations is less than the number of features, the covariance matrices cannot be estimated reliably from the sample. In these cases, within‐set covariance matrices are usually replaced by identity matrices, and sparse CCA is then equivalent to sparse PLS. Please note that researchers may still name this technique as sparse CCA even after this replacement (Witten, Tibshirani, & Hastie, 2009).

With known prior information about features or observations, sparse CCA can be further modified to *structure sparse CCA* or *discriminant sparse CCA*, respectively. If the known prior information is about features, such as categorizing features into different groups (Lin et al., 2014) or characterizing connections between features (Kim et al., 2019), the prior information will be implemented as an additional penalty on features, leading to *structure sparse CCA*. Alternatively, if the known prior information is about observations, such as diagnostic group of each subject, the prior information will be implemented as additional constraint on observations, leading to *discriminant sparse CCA* (Wang et al., 2019).

###### Solutions

Sparse CCA, structure sparse CCA, and discriminant sparse CCA can all be considered as special cases of a generalized constrained CCA (Equation (6)) problem with different equality and inequality constraint sets. Iterative optimization techniques used to solve the generalized constrained CCA problem are also applicable here to solve these special cases.

#### 3.2.2 Nonlinear CCA

Both CCA and constrained CCA assume linear intervariable relationships, however, this assumption does not hold in general for all variables in real data. Nonlinear CCA uncovers the joint nonlinear relationship between different variables, which is a complementary tool to conventional CCA methods. Kernel CCA, temporal kernel CCA, and deep CCA are the foremost techniques in this category.

##### Kernel CCA and temporal kernel CCA

###### Formulation

Kernel CCA uncovers the joint nonlinear relationship between two variables by mapping the original feature space in ***Y***_1_ and ***Y***_2_ on to a new feature space through a *predefined kernel function*. However, this new feature space is not explicitly defined. Instead, the original feature space for each observation in ***Y***_*k*_ is implicitly projected to a higher dimensional feature space ***Y***_*k*_ → *ϕ*(***Y***_*k*_) embedded in a prespecified kernel function $H_{k}\;\in \;R^{N\times N}$, which is independent of the number of features in the projected space. After transforming ***u***_*k*_ to *ϕ*(***Y***_*k*_)^*T*^***v***_*k*_, the CCA form in Equation (1) in the higher dimensional feature space, namely kernel CCA can be written as:

(8) $$\begin{array}{c} \text{Kernel}\;\mathrm{CCA}:\max_{u_{1},u_{2}}\rho =\text{corr}\left(\phi \left(Y_{1}\right)u_{1},\phi \left(Y_{2}\right)u_{2}\right)=\max_{v_{1},v_{2}}\frac{v_{1}^{T}H_{1}H_{2}v_{2}}{\sqrt{v_{1}^{T}H_{1}^{2}v_{1}}\sqrt{v_{2}^{T}H_{2}^{2}v_{2}}};\\ \text{where}\;H_{k}=\mathrm{dot}\;\left(\phi \left(Y_{k}\right),\phi \left(Y_{k}\right)\right)\in R^{N\times N}\text{and}\;u_{k}=\phi {\left(Y_{k}\right)}^{T}v_{k},k=1,2, \end{array}$$

where ***v***_1_ and ***v***_2_ are unknowns to estimate, instead of ***u***_1_ and ***u***_2_.

Temporal kernel CCA is a kernel CCA variant that is specifically designed for two time series with temporal delays. In temporal kernel CCA, one variable, for example, ***Y***_1_, is shifted for multiple different time points and a new variable ${\tilde{Y}}_{1}$ is formed by concatenating the original ***Y***_1_ and the temporally shifted ***Y***_1_. The new variable ${\tilde{Y}}_{1}$ and the original ***Y***_2_ are then input to kernel CCA as in Equation (8).

###### Solution

Closed‐form analytical solution exists for kernel CCA (Equation (8)). By setting the partial derivatives of the objective function in Equation (8) with respect to ***v***_1_ and ***v***_2_ to zero separately, kernel CCA can be converted to the following problem:

(9) $$H_{1}H_{2}v_{2}=\rho H_{1}^{2}v_{1}\;\text{and}\;H_{2}H_{1}v_{1}=\rho H_{2}^{2}v_{2}.$$

Note that the kernel CCA problem defined in Equation (9) always holds true when *ρ* = 1. To avoid this trivial solution, a penalty term needs to be introduced to the norm of original canonical coefficients ***u***_*k*_, such that $v_{k}^{T}H_{k}^{2}v_{k}$ become $v_{k}^{T}H_{k}^{2}v_{k}+\lambda {\left\|u_{k}\right\|}^{2}=v_{k}^{T}\left(H_{k}^{2}+\lambda H_{k}\right)v_{k}$, where *λ* is a regularization parameter. This regularized kernel CCA problem can be further represented as an eigenvalue problem (Hardoon, Szedmak, & Shawe‐Taylor, 2004):

(10) $$\begin{array}{c} {\left(H_{1}+\lambda I\right)}^{-1}H_{2}{\left(H_{2}+\lambda I\right)}^{-1}H_{1}v_{1}=\rho^{2}v_{1}\\ {\left(H_{2}+\lambda I\right)}^{-1}H_{1}{\left(H_{1}+\lambda I\right)}^{-1}H_{2}v_{2}=\rho^{2}v_{2} \end{array},$$

where a closed‐form solution exists in the new feature space.

##### Deep CCA

###### Formulation

Kernel CCA requires a predefined kernel function for the feature mapping to uncover the joint nonlinear relationship between two variables. Alternatively, recent development of deep learning makes it possible to learn the feature mapping from data itself. The deep learning variant of CCA, deep CCA (Andrew, Bilmes, & Livescu, 2013), provides a more flexible and robust way to learn and search the nonlinear association between two variables. More specifically, deep CCA first passes the original ***Y***_1_ and ***Y***_2_ through multiple stacked layers of nonlinear transformations. Let ***θ***_1_ and ***θ***_2_ represent vectors of all parameters through all layers for ***Y***_1_ and ***Y***_2_, respectively, deep CCA can be represented as:

(11) $$\text{Deep}\;\mathrm{CCA}:\max_{\theta_{1},\theta_{2}}\rho =\text{corr}\left(f\left(Y_{1};\theta_{1}\right),f\left(Y_{2};\theta_{2}\right)\right).$$

###### Solution

Deep CCA is solved through a deep learning schema by dividing the original data into training and testing sets. ***θ***_1_ and ***θ***_2_ are optimized by following the gradient of the correlation objective as estimated on the training data (Andrew et al., 2013). The number of unknown parameters in deep CCA is much higher than the number of unknowns in other CCA variants; therefore, a large number of training samples (in tens of thousands) are required for deep CCA to produce meaningful results. In most studies, it is unlikely to have enough observations (e.g. subjects) as training samples for deep CCA algorithms. Instead, in neuroscience applications, treating each brain voxel as a training sample, similar to Yang et al. (2020, 2019), would be more promising in deep CCA applications.

#### 3.2.3 Multiset CCA

Multiset CCA extends the conventional CCA from uncovering associations between two variables to finding common patterns among more than two variables. Constraints can also be incorporated in multiset CCA for various purposes.

##### Multiset CCA

###### Formulation

The most intuitive formulation of multiset CCA is to optimize canonical coefficients of all variables by maximizing pairwise canonical correlations, nameed as SUMCOR multiset CCA:

(12) $$\text{SUMCOR multiset}\;\mathrm{CCA}:\max_{u_{1},\ldots ,u_{K}}\sum_{i,j,i\neq j}^{K}\text{corr}\left(Y_{i}u_{i},Y_{j}u_{j}\right),$$

where *K* > 2 is the number of variables. A new matrix $\hat{\sum }\in R^{K\times K}$ is defined where each element ${\hat{\sum }}_{i,j}\;$ is a canonical correlation between two variables ***Y***_*i*_ and ***Y***_*j*_:

(13) $$\hat{\sum }=\left[\begin{array}{ccc} \begin{array}{cc} u_{1}^{T}\sum_{11}u_{1} & u_{1}^{T}\sum_{12}u_{2}\\ u_{2}^{T}\sum_{21}u_{1} & u_{2}^{T}\sum_{22}u_{2} \end{array} & \cdots & \begin{array}{c} u_{1}^{T}\sum_{1K}u_{K}\\ u_{2}^{T}\sum_{2K}u_{K} \end{array}\\ \vdots & \ddots & \vdots \\ u_{K}^{T}\sum_{K1}u_{1}\;u_{K}^{T}\sum_{K2}u_{2} & \cdots & u_{K}^{T}\sum_{\mathrm{KK}}u_{K} \end{array}\right],$$

and $u_{k}^{T}\sum_{\mathrm{kk}}u_{k},k=1,\ldots ,K$ is set to 1 for normalization.

Besides maximizing SUMCOR, Kettenring (1971) summarizes four other possible objective functions in multiset CCA optimization: (a) SSQCOR, maximizing sum of squared pairwise correlations ${\sum_{i,j}^{K}\hat{\sum }}_{\mathrm{ij}}^{2}$; (b) MAXVAR, maximizing largest eigenvalue of correlation matrix $\lambda_{\max}\left(\hat{\sum }\right)$; (c) MINVAR, minimizing smallest eigenvalue of correlation matrix $\lambda_{\min}\left(\hat{\sum }\right)$; and (d) GENVAR, minimizing the determinant of correlation matrix $\det\left(\hat{\sum }\right)$. In practice, SUMCOR multiset CCA is most commonly used followed by MAXVAR and SSQCOR multiset CCA.

###### Solution

Analytical solutions of multiset CCA are obtained by calculating the partial derivatives of the objective function with respect to each ***u***_*i*_. Since SUMCOR and SSQCOR are linear and quadratic functions of each ***u***_*i*_, respectively, closed‐form analytical solutions can be obtained for these two cost functions by setting the partial derivatives equal to 0, which leads to generalized eigenvalue problems. Multiset CCA with all these five objective functions can also be solved by means of the general algebraic modeling system (Brooke, Kendrick, Meeraus, & Rama, 1998) and NLP solver CONOPT (Drud, 1985).

##### Multiset CCA with constraints

In constrained multiset CCA, penalty terms can be added to each ***u***_*i*_ individually. Here we give examples of two commonly incorporated constraints in multiset CCA: sparse multiset CCA and multiset CCA with reference.

###### Formulation: Sparse multiset CCA

Similar to sparse CCA, sparse multiset CCA applies the *L*_1_‐norm penalty to one or more ***u***_*i*_ in Equation (12), and therefore induces sparsity on canonical coefficient(s) and can be applied to high‐dimensional variables. Here, we give the equation of SUMCOR sparse multiset CCA as an example:

(14) $$\text{SUMCOR sparse multiset}\;\mathrm{CCA}:\max_{u_{1},\ldots ,u_{K}}\sum_{i,j,i\neq j}^{K}\text{corr}\left(Y_{i}u_{i},Y_{j}u_{j}\right),\;s.t.{\left|u_{i}\right|}_{1}<c_{i}.$$

###### Formulation: Multiset CCA with reference

Multiset CCA with reference enables the discovery of multimodal associations with a specific reference variable across subjects, such as a neuropsychological measurement (Qi, Calhoun, et al., 2018). In multiset CCA with reference, additional constraints of correlations between each canonical variable and the reference variable (***v***_ref_) are added:

(15) $$\text{SUMCOR multiset}\;\mathrm{CCA}\;\text{with}\;\mathrm{ref}:\max_{u_{1},\ldots ,u_{K}}\sum_{i,j,i\neq j}^{K}\left(\text{corr}\left(Y_{i}u_{i},Y_{j}u_{j}\right)+\lambda \;{\left|\left|\text{corr}\left(Y_{i}u_{i},v_{\mathrm{ref}}\right)\right|\right|}_{2}^{2}\right),$$

where *λ*>0 is the tuning parameter and ${\left||∙|\right|}_{2}^{2}$ is the *L*_2_‐norm. Therefore, multiset CCA with reference is a *supervised* multivariate technique that can extract common components across multiple variables that are associated with a specific prior reference.

###### Solution

Both Equations (14) and (15) can be viewed as constrained optimization problems with an objective function and multiple equality and inequality constraints. In this case, iterative optimization techniques are required to solve constrained multiset CCA problems.

#### 3.2.4 Other CCA‐related techniques

There are many other CCA‐related techniques developed, and here we only included three that have been applied in the neuroscience field: supervised local CCA, Bayesian CCA, and tensor CCA.

##### Supervised local CCA

CCA by formulation is an unsupervised technique that uncovers joint relationships between two variables. Meanwhile, CCA can become a supervised technique by (a) adding additional constraints such as CCA (multiset CCA) with reference discussed in the section “Multiset CCA with constraints,” or (b) directly incorporating group information into the objective function as in the *supervised local CCA* technique (Zhao et al., 2017).

Supervised local CCA is based on locally discriminant CCA (Peng, Zhang, & Zhang, 2010), which uses local group information to construct a between‐set covariance matrix ${\tilde{\sum }}_{12}$, as a replacement of **∑**_12_ in Equation (1). More specifically, ${\tilde{\sum }}_{12}$ is defined as the covariance matrix from *d* nearest neighboring *within‐class* samples (**∑**_*w*_) penalized by the covariance from *d* nearest neighboring *between‐class* samples (**∑**_*b*_) with a tuning parameter *λ*,

(16) $${\tilde{\sum }}_{12}=\sum_{w}-\lambda \sum_{b}.$$

However, this technique only considers the local group information with the global discriminating information ignored. To address this issue, Fisher discrimination information together with local group information is considered in supervised local CCA, which can be written as:

(17) $$\text{Supervised local}\;\mathrm{CCA}:\max_{u_{1},u_{2}}\rho =\frac{u_{1}^{T}{\tilde{\sum }}_{12}u_{2}+u_{1}^{T}S_{1}u_{1}+u_{2}^{T}S_{2}u_{2}}{\sqrt{u_{1}^{T}\sum_{11}u_{1}}\sqrt{u_{2}^{T}\sum_{22}u_{2}}},$$

$$S_{k}=Y_{k}^{T}UY_{k},k=1,2,U\in R^{N\times N},$$

where ***S***_*k*_ denote the between‐group scatter matrices of the dataset *k*. If samples *i* and *j* belong to *c*th class, *U*_*ij*_ is set to $\frac{1}{n_{c}}$, where *n*_*c*_ denotes the number of samples in *c*th class; otherwise, *U*_*ij*_ is set to 0. Supervised local CCA is usually applied sequentially with gradually decreased *d* (named as hierarchical supervised local CCA) to reduce the influence of the neighborhood size and improve classification performance.

##### Bayesian CCA

Bayesian CCA is another technique that overcomes the overfitting problem when applying CCA to variables with small sample sizes. Bayesian CCA is also proposed to complement CCA by providing a principal component analysis (PCA)‐like description of variations that are not captured by the correlated components (Klami, Virtanen, & Kaski, 2013). Input to CCA in Equation (1), ***Y***_1_ and ***Y***_2_, can be considered as *N* observations of one‐dimensional random variables $y_{1}\in R^{p_{1}\times 1}$ and $y_{2}\in R^{p_{2}\times 1}$. Using the same notations, Bayesian CCA can be formulated as a latent variable model (with latent variable ***z***) between ***y***_1_ and ***y***_2_ (Klami & Kaski, 2007; Wang, 2007):

(18) $$\begin{array}{c} \mathrm{z∼}N\left(0,I\right),\\ \;\\ \;y_{k}∼N\left(A_{k}z+B_{k}z_{k},D_{k}\right),k=1,2, \end{array}$$

where $N\left(0,I\right)$ denotes the multivariate Gaussian distribution with mean vector **0** and identity covariance matrix ***I***. ***D***_*k*_ are diagonal covariance matrices and indicate features in ***y***_*k*_ with independent noise. The latent variable $z\;\in \;R^{q\times 1}$, where *q* represents the number of shared components, captures the shared variation between ***y***_1_ and ***y***_2_, and can be linearly transformed back to the original space of ***y***_*k*_ through ***A***_*k*_***z*,***k* = 1, 2. Similarly, the latent variable, where *q*_*k*_ represents the number of variable‐specific components, captures the variable *k*‐specific variation not shared between ***y***_1_ and ***y***_2_, and can be linearly transformed back to the original space in ***y***_*k*_ by ***B***_*k*_***z***_*k*_.

Browne (1979) demonstrated that Equation (18) was equivalent to CCA in Equation (1) by showing that maximum likelihood solutions to both Equations (1) and (18) share the same canonical coefficients with an unknown rotational transform, that is, Equation (18) is equivalent to conventional CCA (Equation (1)) in the aspect that their solutions share the same subspace. However, unlike conventional CCA (Equation (1)) that uses two variables ***u***_1_ and ***u***_2_ to project ***y***_1_ and ***y***_2_ to this subspace, Bayesian CCA maintains the shared variation between ***y***_1_ and ***y***_2_ in a single variable ***z***.

The formulation of ***y***_*k*_ in Equation (18) can be rewritten as $y_{k}∼N\left(A_{k}z,B_{k}B_{k}^{T}+D_{k}\right),k=1,2$ after algebra operations. With $\Psi_{k}=B_{k}B_{k}^{T}+D_{k}$, the model in Equation (18) can be transformed to

(19) $$\begin{array}{c} \mathrm{z∼}N\left(0,I\right),\\ \;\\ \;y_{k}∼N\left(A_{k}z,\Psi_{k}\right),k=1,2. \end{array}$$

In Equation (19), prior knowledge of the parameters (e.g., ***A***_*k*_ and **Ψ**_*k*_) are required to construct the latent variable model for Bayesian CCA. For instance, the inverse Wishart distribution as a prior for the covariance **Ψ**_*k*_ and the automatic relevance determination (ARD; Neal, 2012) prior for the linear mappings ***A***_*k*_ are used when Bayesian CCA is proposed (Klami & Kaski, 2007; Wang, 2007). Since then, multiple Bayesian inference techniques have been developed, however, the early work of Bayesian CCA is limited to low‐dimensional data (not more than eight dimensions in Klami & Kaski, 2007 and Wang, 2007) due to the computational complexity to estimate the posterior distribution over the *p*_*k*_ × *p*_*k*_ covariance matrices **Ψ**_*k*_ (Klami et al., 2013). A group‐wise ARD prior (Klami et al., 2013) was recently introduced for Bayesian CCA, which automatically identifies variable‐specific and shared components. More importantly, this change made Bayesian CCA applicable for high‐dimensional data. More technical details about Bayesian CCA can be found in Klami et al. (2013).

##### Tensor CCA

###### Two‐dimensional CCA and tensor CCA for high‐dimensional variables

Variables input to CCA ($Y_{k}\in R^{N\times p_{k}},k=1,2,\ldots ,$) are usually required to be 2D matrices with a dimension of number of observations (*N*) times number of features (*p*_*k*_) in each variable. ***Y***_*k*_ can be considered as *N* observations of the 1D variable $y_{k}\in R^{p_{k}\times 1}$. In practice, tensor data, such as 3D images or 4D time series, are commonly involved in neuroscience applications, and these variables are required to be vectorized before inputting to CCA algorithms. This vectorization could potentially break the feature structures. In this case, to analyze 3D data, such as *N* samples of 2D variables (*N* × *p*_1_ × *p*_2_), without breaking the 2D feature structure, two‐dimensional CCA (2DCCA) has been proposed by Lee and Choi (2007).

Mathematically, 2DCCA maximizes the canonical correlation between two variables with N observations of 2D features: $Y_{1}:\left\{Y_{1}^{n}\in R^{p_{11}\times p_{12}},n=1,\ldots ,N\right\}$ and $Y_{2}:\left\{Y_{2}^{n}\in R^{p_{21}\times p_{22}},n=1,\ldots ,N\right\}$. For each variable, 2DCCA searches left transforms $\;l_{1}\in R^{p_{11}\times 1}$ and $l_{2}\in R^{p_{21}\times 1}$ and right transforms $r_{1}\in R^{p_{12}\times 1}$ and $r_{2}\in R^{p_{22}\times 1}$ in order to maximize the correlation between $l_{1}^{T}Y_{1}r_{1}$ and $l_{2}^{T}Y_{2}r_{2}$:

(20) $$2D\;\mathrm{CCA}:\max_{l_{1},l_{2},r_{1},r_{2}}\rho =\mathrm{cov}\left(l_{1}^{T}Y_{1}r_{1},l_{2}^{T}Y_{2}r_{2}\right),s.t.\mathrm{var}\left(l_{1}^{T}Y_{1}r_{1}\right)=1,\mathrm{var}\left(l_{2}^{T}Y_{2}r_{2}\right)=1.$$

In Equation (20), for fixed ***l***_1_ and ***l***_2_, ***r***_1_ and ***r***_2_ can be obtained with the SVD algorithm similar to the one used in conventional CCA, and ***l***_1_ and ***l***_2_ can be obtained for fixed ***r***_1_ and ***r***_2_, alternatingly. Therefore, an iterative alternating SVD algorithm (Lee & Choi, 2007) has been developed to solve Equation (20).

Above described 2DCCA can be treated as a constrained optimization problem with low‐rank restrictions on canonical coefficients, similar restrictions are used in (Chen, Kolar, & Tsay, 2019), where 2DCCA has been extended to higher dimensional tensor data, termed tensor CCA. The tensor CCA (Chen et al., 2019) searches two rank‐one tensors $u_{1}=u_{11}\circ \cdots \circ u_{1m}\in R^{p_{11}\times \cdots \times p_{1m}}$ and $u_{2}=u_{21}\circ \cdots \circ u_{2m}\in R^{p_{21}\times \cdots \times p_{2m}}$ to maximize the correlation between $Y_{1}:\left\{Y_{1}^{n}\in R^{p_{11}\times \cdots \times p_{1m}},n=1,\ldots ,N\right\}$ and $Y_{2}:\left\{Y_{2}^{n}\in R^{p_{21}\times \cdots \times p_{2m}},n=1,\ldots ,N\right\}$, where “∘” denotes outer product and ***u***_*k*1_, …, ***u***_*km*_ are vectors. Chen et al. (2019) also introduced an efficient optimization algorithm to solve tensor CCA for high dimensional data sets.

###### Tensor CCA for multiset data

Another way to handle input variables with high‐dimensional feature spaces is to generalize conventional CCA by analyzing constructed covariance tensors (Luo, Tao, Ramamohanarao, Xu, & Wen, 2015). This method requires random variables to be vectorized and is similar to multiset CCA since both of them deal with more than two input modalities. The differences between tensor CCA and multiset CCA in this case lie in that tensor CCA constructs a high‐order covariance tensor for all input variables (Luo et al., 2015), whereas multiset CCA finds pair‐wise covariance matrices. In addition, tensor CCA (Luo et al., 2015) does not maximize the pairwise correlation as in multiset CCA; instead, it directly maximizes the correlation over all canonical variables,

(21) $$\max_{u_{1},\ldots ,u_{K}}\rho =\text{Corr}\left(Y_{1}u_{1},\cdots ,Y_{K}u_{K}\right)={\left(Y_{1}u_{1}ʘ\cdots ʘY_{K}u_{K}\right)}^{T}\times 1;s.t.{\left(Y_{k}u_{k}\right)}^{T}Y_{k}u_{k}=1,k=1,\ldots ,K,$$

where ʘ denotes element‐wise product and $1\;\in \;R^{N\times 1}$ is an all ones vector. The problem formulated in Equation (21) can be solved by using the alternating least square algorithm (Kroonenberg & de Leeuw, 1980).

#### 3.2.5 Statistical inferences of CCA variants

Nonparametric permutation tests have been widely performed in CCA variant techniques to determine the statistical significance of each canonical correlation value and the corresponding canonical coefficients. In these permutation tests, as we described in Section 3.1, observations of one variable are randomly shuffled (***Y***_1_ becomes $\hat{Y_{1}}$), while observations of the other variable are kept intact (***Y***_2_ remains). This random shuffling is repeated multiple times (~5,000), and the exact same CCA variant technique is applied to each shuffled data. The obtained canonical correlation values from these randomly shuffled data form the null distribution. Statistical significances (*p*‐values) of true canonical correlation values are determined by comparing true values to this null distribution.

Besides permutation tests, a null distribution can also be built by creating null data input to CCA variant techniques. The null data are usually generated based on the physical properties of input variables. For instance, when applying CCA‐variant technique to link task fMRI data and the task stimuli, the null data of task fMRI can be obtained by applying wavelet‐resampling to resting‐state fMRI data (Breakspear, Brammer, Bullmore, Das, & Williams, 2004; Zhuang et al., 2017). The null hypothesis here is that task fMRI data are not multivariately correlated with task stimuli, and the wavelet resampled resting‐state fMRI data fits the requirements of the null data in this case.

### 3.3 Technical differences

#### 3.3.1 Technical differences among CCA‐related techniques

There are three prominent CCA techniques: conventional CCA shares the simplest formulation and can be easily applied to uncover multivariate linear relationships between two variables; nonlinear CCA by definition can extract multivariate nonlinear relationship between two variables through feature mapping with known predefined functions; and multiset CCA are able to find common covariated patterns among more than two variables. These three methods can be efficiently solved with closed‐form analytical solutions, which are obtained by taking the partial derivatives of the objective function with respective to each unknown, separately.

Constrained (multiset) CCA incorporates prior information about input variables into each of the three CCA methods, in terms of equality and inequality constraints on the unknowns. Prior knowledge about the data or specific hypothesis are required for its applications. Closed‐form solutions are no longer available for constrained (multiset) CCA and iterative optimization techniques are required to solve these problems.

Recently developed deep CCA is different from all other CCA‐related techniques as it learns the optimum feature mapping from the data itself through deep learning with training and testing data being specified. Machine learning and deep leaning expertise are required to solve this problem.

#### 3.3.2 Relationship between CCA and other multivariate and univariate techniques

##### Relationship with other multivariate techniques

In general, CCA can be directly rewritten in terms of the multivariate multiple regression (MVMR) model:

(22) $$Y_{1}u_{1}=Y_{2}u_{2}+\varepsilon ,$$

where ***u***_1_ and ***u***_2_ are obtained by minimizing the residual term $\varepsilon \;\in \;R^{N\times 1}$. Since CCA is scale‐invariant, a solution to Equation (22) is also a solution of Equation (1). Furthermore, with normalization terms of $u_{1}^{T}\sum_{11}u_{1}=1$ and $u_{2}^{T}\sum_{22}u_{2}=1,$ the MVMR model is exactly equivalent to CCA, that is, maximizing the canonical correlation between ***Y***_**1**_ and ***Y***_**2**_ is equivalent to minimizing the residual term ***ε***:

(23) $$\max_{u_{1},u_{2}}\left(\text{corr}\left(Y_{1}u_{1},Y_{2}u_{2}\right)\right)\Leftrightarrow \max_{u_{1},u_{2}}u_{1}^{T}\sum_{12}u_{2}\Leftrightarrow \min_{u_{1},u_{2}}-u_{1}^{T}\sum_{12}u_{2}\Leftrightarrow \min_{u_{1},u_{2}}{\left|\left|Y_{1}u_{1}-Y_{2}u_{2}\right|\right|}_{2}^{2}.$$

In addition, by replacing the covariance matrices **∑**_11_ and **∑**_22_ in the denominator in Equation (1) with the identity matrix ***I***, conventional CCA is converted to partial least square (PLS), which maximizes the covariance between latent variables. If ***Y***_1_ is the same as ***Y***_2_, the PLS will maximize the variance within a single variable, which is equivalent to PCA.

##### Relationship with univariate techniques

If one variable in CCA, for example, ***Y***_1_, only has a single feature, that is, $y\;\in \;R^{N\times 1}$, ***u***_1_ can then be defined as 1 and CCA becomes a linear regression problem:

(24) y=Xβ+ε,

where ***Y***_1_ is renamed as ***y*** and ***Y***_2_ is renamed as ***X*** to follow conventional notations. $\varepsilon \;\in \;R^{N\times 1}$ denotes the residual term. If both variables ***Y***_1_ and ***Y***_2_ contain only one feature, the canonical correlation between ***Y***_1_ and ***Y***_2_ becomes the Pearson's correlation between ***Y***_1_ and ***Y***_2_ as in the univariate analysis.

## 4 NEUROSCIENCE APPLICATIONS

### 4.1 CCA: Finding linear relationships

#### 4.1.1 Direct application of CCA

##### Combine phenotypes and brain activities

To date, the most common CCA application in neuroscience is to find joint multivariate linear associations between phenotypic features and neurobiological activities. Phenotypic features usually include one or more measurements from demographics, genetic information, behavioral measurements, clinical symptoms, and performances of neuropsychological tests. Neurobiological activities are generally summarized with brain structural measurements, functional activations during specific tasks, both static and dynamic resting‐state functional connectivity measurements, network topological measurements, and electrophysiological recordings (Table 1).

**TABLE 1:** CCA application

CCA variant	Modality 1	Modality 2	References
CCA	Brain imaging data	Clinical/behavioral/neuropsychological measurements	Adhikari et al. (2019); Chenausky, Kernbach, Norton, and Schlaug (2017); Drysdale et al. (2017); Kottaram et al. (2019); Kucukboyaci et al. (2012); Kuo, Kutch, and Fisher (2019); Liao et al. (2010); Lin, Cocchi, et al. (2018); Lin, Vavasour, et al. (2018); Palaniyappan et al. (2019); Rodrigue et al. (2018); Shen et al. (2016); Tian, Zalesky, Bousman, Everall, and Pantelis (2019); Tsvetanov et al. (2016); Wee et al. (2017)
	Brain imaging data	Brain imaging data	Ashrafulla et al. (2013); Brier et al. (2016); Irimia and van Horn (2013); Li et al. (2017); Liu et al. (2018); Neumann et al. (2006); Palaniyappan et al. (2019); Viviano et al. (2018); Zhu, Suk, Lee, and Shen (2016)
	Brain imaging data	Task design	El‐Shabrawy et al. (2007); Nandy and Cordes (2003); Nandy and Cordes, (2004); Rydell, Knutsson, and Borga (2006); Shams, Hossein‐Zadeh, and Soltanian‐Zadeh (2006)
	Electrophysiological data	Clinical/behavioral measurements	Abraham et al. (1996)
	Electrophysiological data	Electrophysiological data	Brookes et al. (2014) (windowed‐CCA), Ji (1999), McCrory and Ford (1991), Somers and Bertrand (2016), and Soto et al. (2016)
	Electrophysiological data	Stimulus	de Cheveigne et al. (2018); Dmochowski, Ki, DeGuzman, Sajda, and Parra (2018)
	Genetic information	Clinical/behavioral measurements	Laskaris et al. (2019); Kim, Won, Youn, and Park (2019);
	Clinical/behavioral/demographics/neuropsychological measurements	Clinical/behavioral/demographics/neuropsychological measurements	Bedi et al. (2015); Dell'Osso et al. (2014); Gulin et al. (2014); Leibach, Stern, Arelis, Islas, and Barajas (2016); Lin et al. (2017); Lin, Cocchi, et al. (2018); Lin, Vavasour, et al. (2018); Lopez et al. (2017); Mirza et al. (2018); Valakos et al. (2018); Will et al. (2017)
	Blind‐source separation to denoise electrophysiological data		Hallez et al. (2009); Janani et al. (2020); von Luhmann, Boukouvalas, Muller, and Adali (2019); Vergult et al. (2007)
PCA/LASSO/regression + CCA	Brain imaging data	Clinical/behavioral/neuropsychological measurements	Churchill et al. (2012); Hackmack et al. (2012); Li et al. (2019); Mihalik et al. (2019); Smith et al. (2015); Zarnani et al. (2019)
	Brain imaging data	Brain imaging data	Abrol, Rashid, Rachakonda, Damaraju, and Calhoun (2017); Hirjak et al. (2019); Ouyang et al. (2015); Yang, Cao, et al. (2018); Yang, Zhuang, et al. (2018); Sato et al. (2010); Sui et al. (2010, 2011)
	Brain imaging data	Genetic data	Bai, Zille, Hu, Calhoun, and Wang (2019); Zille, Calhoun, and Wang (2018)
	Electrophysiological data	Clinical/behavioral measurements	Bologna et al. (2018)

Abbreviations: CAA, canonical correlation analysis; LASSO, least absolute shrinkage and selection operator; PCA, principal component analysis.

In normal healthy subjects, using CCA, multiple studies have delineated the joint multivariate relationships between the above imaging‐derived features and nonimaging measurements, which have boosted our understandings of healthy development and healthy aging (Irimia & van Horn, 2013; Kuo et al., 2019; Shen et al., 2016; Tsvetanov et al., 2016). Furthermore, using multivariate CCA to combine imaging and nonimaging features have provided new insights to understand the joint relationship between brain activities and subjects' clinical symptoms, behavioral measurements, and performances of neuropsychological tests in various diseased populations, such as psychosis disease spectrum (Adhikari et al., 2019; Bai et al., 2019; Kottaram et al., 2019; Laskaris et al., 2019; Palaniyappan et al., 2019; Rodrigue et al., 2018; Tian et al., 2019; Viviano et al., 2018), Alzheimer's disease spectrum (Brier et al., 2016; Liao et al., 2010; McCrory & Ford, 1991; Zhu et al., 2016), neurodevelopmental diseases (Chenausky et al., 2017; Lin, Cocchi, et al., 2018; Zille et al., 2018), depression (Dinga et al., 2019), Parkinson's disease (Lin, Baumeister, Garg, and McKeown, 2018; Liu et al., 2018), multiple sclerosis (Leibach et al., 2016; Lin et al., 2017), epilepsy (Kucukboyaci et al., 2012) and drug addictions (Dell'Osso et al., 2014).

##### Brain activation in response to task stimuli

CCA has also been applied to detect brain activations in responses to stimuli during task‐based fMRI experiments. Compared to the most commonly general linear regression model, local neighboring voxels are considered simultaneously in CCA to determine activation status of the central voxel (Friman, Cedefamn, Lundberg, Borga, & Knutsson, 2001; Nandy & Cordes, 2003; Nandy & Cordes, 2004; Rydell et al., 2006; Shams et al., 2006). In addition, in task‐based electrophysiological experiments, Dmochowski et al. (2018) and de Cheveigne et al. (2018) have maximized the canonical correlation between an optimally transformed stimulus and properly filtered neural responses to delineate the stimulus–response relationship in electroencephalogram (EEG) data.

##### Denoising neuroscience data

Another application of CCA in neuroscience research is to remove noises from signals in the raw data. Through a blind source separation (BSS) framework, von Luhmann et al. (2019) extract comodulated canonical components between fNIRS signals and accelerometer signals, and consider those components above a canonical correlation threshold to be motion artifact. Through BSS‐CCA algorithms, multiple studies demonstrate that muscle artifact can be efficiently removed from EEG signals (Hallez et al., 2009; Janani et al., 2020; Somers & Bertrand, 2016; Vergult et al., 2007). Furthermore, Churchill et al. (2012) remove physiological noise from fMRI signals through a CCA‐based split‐half resampling framework, and Li et al. (2017) remove gradient artifacts in concurrent EEG/fMRI recordings through maximizing the temporal autocorrelations of the time series.

##### Canonical granger causality

CCA has also been used to determine the causal relationship among regions of interest (ROIs) in fMRI functional connectivity analysis. Instead of using the mean ROI time series directly for analysis, multiple time series are specified for each ROI and CCA searches the optimally weighted mean time series during the analysis. Sato et al. (2010) compute multiple eigen‐time series for each ROI and determine the granger causality between two ROIs by maximizing the canonical correlation between eigen‐time series at time point t and t‐1 of the two ROIs. In a more recent work, instead of using eigen‐time series of each ROI, Gulin et al. (2014) compute an optimized linear combination of signals from each ROI in CCA to enable a more accurate causality measurement.

#### 4.1.2 Practical considerations and data reduction steps

As we stated in Section 3.1, only if numbers of observations are more than numbers of features in both ***Y***_1_ and ***Y***_2_, that is, *N* ≫ *p*_*k*_, *k* = 1, 2, conventional CCA can produce statistically stable and meaningful results. However, in neuroscience applications, this requirement is not always fullfilled, especially when ***Y***_1_ or ***Y***_2_ represents brain activities where each brain voxel is considered a feature individually. In this case, any feature can be picked up and learned by the CCA process and directly applying Equation (1) to two sets will produce overfitted and unstable results. Therefore, additional data‐reduction steps applied before CCA or constraints incorporated in the CCA algorithm are necessary to avoid overfitting in CCA applications. In this section, we focus on data reduction steps applied before conventional CCA.

The most commonly used data reduction technique is the PCA method applied to ***Y***_1_ and ***Y***_2_ separately. Through orthogonal transformation, PCA converts ***Y***_1_ and ***Y***_2_ into sets of linearly uncorrelated principal components. The principal components that do not pass certain criteria are discarded, leading to dimension‐reduced variables: ${\tilde{Y}}_{1}\in R^{N\times q_{1}}$ and ${\tilde{Y}}_{2}\in R^{N\times q_{2}}$, where *N* ≫ *q*_*k*_, *k* = 1, 2. Equation (1) can then be applied to ${\tilde{Y}}_{1}$ and ${\tilde{Y}}_{2}$. Multiple studies applied PCA to reduce data dimensions before applying CCA to find joint multivariate correlations between two high‐dimensional variables (Abrol et al., 2017; Churchill et al., 2012; Hackmack et al., 2012; Li et al., 2019; Mihalik et al., 2019; Ouyang et al., 2015; Sato et al., 2010; Smith et al., 2015; Sui et al., 2010; Sui et al., 2011; Zarnani et al., 2019).

In addition, the least absolute shrinkage and selection operator (LASSO) algorithm (Tibshirani, 1996) has also been applied prior to CCA as a feature selection step to eliminate less informative features. For instance, in delineating the association between neurophysiological measures, which are derived from transcranial magnetic stimulation and electromyographic recordings, and kinematic‐clinical‐demographic measurements in Parkinson's disease subjects, Bologna et al. (2018) first perform logistic regression with LASSO penalty to determine the most predictive features for the disease in both variables. CCA is then applied to link the most predictive features from each variable. Similarly, sparse regression techniques have also been applied before CCA to genetic data in a neurodevelopmental cohort (Zille et al., 2018). Furthermore, feature selection can also be implemented in PCA as done in *L*_1_‐norm penalized sparse PCA (sPCA; Witten & Tibshirani, 2009; Yang, Zhuang, Bird, et al., 2019), which removes noninformative features during the dimension reduction step.

There is no single “correct” way or “gold standard” of the feature reduction step before applying CCA. Decisions should be made based on the data itself and the specific question that researchers are interested in.

### 4.2 Constrained CCA: Removing noninformative features and stabilizing results

The other common solution in practice for *N* ≪ *p*_*k*_, *k* = 1, 2 is to incorporate constraints into the CCA algorithm directly, and consequently noninformative features can be removed and overfitting problems can be avoided (Table 2).

**TABLE 2:** Constrained CCA application

CCA variant	Modality 1	Modality 2	Reference
Sparse CCA (L1‐norm penalty)	Brain imaging data	Clinical/behavioral/neuropsychological measurements	Badea et al. (2019); Lee, Moser, Ing, Doucet, and Frangou (2019); Moser et al. (2018); Pustina, Avants, Faseyitan, Medaglia, and Coslett (2018); Thye and Mirman (2018); Vatansever et al. (2017); Wang et al. (2018); Xia et al. (2018)
	Brain imaging data	Brain imaging data	Avants, Cook, Ungar, Gee, and Grossman (2010); Deligianni, Carmichael, Zhang, Clark, and Clayden (2016); Deligianni, Centeno, Carmichael, and Clayden (2014); Duda, Detre, Kim, Gee, and Avants (2013); Jang et al. (2017); Kang, Kwak, Yoon, and Lee (2018); Rosa et al. (2015); Sintini, Schwarz, Martin et al. (2019); Sintini, Schwarz, Senjem, et al. (2019)
	Brain imaging data	Genetic information	Du et al. (2016); Du, Liu, Yao, et al. (2019); Du, Liu, Zhu, et al. (2019); Grellmann et al. (2015); Gossmann, Zille, Calhoun, and Wang (2018); McMillan et al. (2014); Sheng et al. (2014); Szefer, Lu, Nathoo, Beg, and Graham (2017); Wan et al. (2011)
	Genetic information	Clinical/behavioral/measurements	Leonenko et al. (2018)
Structure‐sparse CCA	Brain imaging data	Brain imaging data	Lisowska and Rekik (2019); Mohammadi‐Nejad, Hossein‐Zadeh, and Soltanian‐Zadeh (2017)
	Brain imaging data	Genetic information	Du et al. (2014, 2015, 2016a, 2016b; Du et al. (2017); Kim et al. (2019); Liu et al. (2017; Lin, Calhoun, and Wang, 2014; Yan et al. (2014
Discriminant sparse CCA	Brain imaging data	Genetic information/blood data	Fang et al. (2016); Wang, Shao, Hao, Shen, and Zhang (2019); Yan, Risacher, Nho, Saykin, and Shen (2017)
Constrained CCA	Brain imaging data	Clinical/behavioral/neuropsychological measurements	Grosenick et al. (2019); Dashtestani et al. (2019)
	Brain imaging data	Task design	Cordes, Jin, Curran, and Nandy, (2012a, 2012b); Dong et al. (2015); Friman, Borga, Lundberg, and Knutsson (2003); Zhuang et al. (2017); Zhuang et al. (2019)
Other constraints in CCA	Longitudinal brain imaging data	Genetic information	Du, Liu, Zhu, et al. (2019) (temporal multitask sparse CCA); Hao et al. (2017) (temporal group sparse CCA);

Abbreviation: CCA, canonical correlation analysis.

#### 4.2.1 Constraints in CCA algorithms: Sparse CCA to remove noninformative features

Most studies apply the sparse CCA method (detailed in the section “Special case: L_1_‐norm penalty and sparse CCA”), which maximizes canonical correlations between ***Y***_1_ and ***Y***_2_, and suppresses noninformative features in ***Y***_1_ and ***Y***_2_ simultaneously (Badea et al., 2019; Lee et al., 2019; Moser et al., 2018; Pustina et al., 2018; Thye & Mirman, 2018; Vatansever et al., 2017; Wang et al., 2018; Xia et al., 2018). The features determined to be noninformative are assigned with zero coefficients. Therefore, sparse CCA is particularly appropriate to combine modalities with large noise or substantial noninformative features, such as voxel‐wise, regional‐wise or connectivity‐based brain features and genetic sequences (Avants et al., 2010; Deligianni et al., 2014; Du et al., 2017; Du, Liu, Yao, et al., 2019; Du, Zhang, et al., 2016; Duda et al., 2013; Gossmann et al., 2018; Grellmann et al., 2015; Jang et al., 2017; Kang et al., 2018; McMillan et al., 2014; Sheng et al., 2014; Sintini, Schwarz, Martin, et al., 2019; Sintini, Schwarz, Senjem, et al., 2019; Szefer et al., 2017; Wan et al., 2011). Rosa et al. (2015) further induce nonnegativity in the *L*_1_‐norm penalty in sparse CCA to investigate multivariate similarities between the effects of two antipsychotic drugs on cerebral blood flow using collected arterial spin labeling data.

Prior knowledge about ***Y***_1_ and ***Y***_2_ might also be available in neuroscience data. With known prior information of the feature dimension, structure‐sparse CCA has been applied to associate brain activities with genetic information (Du et al., 2014; Du et al., 2015; Du, Huang, et al., 2016a; Du, Huang, et al., 2016b; Du, Liu, Zhang, et al., 2017; Kim et al., 2019; Lin et al., 2014; Liu et al., 2017; Yan et al., 2014), and to link structural and functional brain activities (Lisowska & Rekik, 2019; Mohammadi‐Nejad et al., 2017). If prior knowledge is available of the observation dimension, such as memberships of diagnostic groups, discriminant sparse CCA is applied to investigate joint relationship between brain activities and genetic information for subjects with Schizophrenia disease spectrum (Fang et al., 2016) or Alzheimer's disease spectrum (Wang et al., 2019; Yan et al., 2017). Longitudinal data could also be collected in neuroscience research and are useful to monitor disease progression. Temporal constrained sparse CCA has been proposed to uncover how single nucleotide polymorphisms affect brain gray matter density across multiple time points in subjects with Alzheimer's disease spectrum (Du, Liu, Zhu, et al., 2019; Hao, Li, Yan, et al., 2017).

#### 4.2.2 Constraints in CCA algorithm: Constrained CCA to stabilize results

Multiple constraints have also been proposed in CCA applications to stabilize CCA coefficients between brain activities and clinical symptoms. For instance, to avoid overfitting between fNIRS signals during a moral judgment task and psychopathic personality inventory scores in healthy adults, Dashtestani et al. (2019) introduce a regularization parameter *λ* to keep the canonical coefficients small and to avoid high bias problem. Similarly, in preclinical research, Grosenick et al. (2019) uses two regularization parameters *λ*_1_ and *λ*_2_ to penalize the estimated covariance matrices for the resting‐state functional connectivity features and Hamilton Rating Scale for Depression clinical symptoms, respectively.

Furthermore, as we stated in Section 4.1.1, CCA has been applied to detect brain activations in response to task stimuli during fMRI experiments. In these type of applications, ***Y***_1_ represents time series from local neighborhood that is considered simultaneously in determining the activation status of the central voxels, and ***Y***_2_ represents the task design matrix. CCA is applied to find optimized coefficients ***u***_1_ and ***u***_2_, such that the correlation between combined local voxels and task design is maximized. In this case, even though the central voxel may be inactivated in the task, activated neighboring voxels would lead to a high canonical correlation and thus produce falsely activated status of the central voxel, which is termed assmoothing artifact (Cordes et al., 2012a). To eliminate this artifact and to uncover real activation status, multiple constraints have been applied to ***u***_1_ to guarantee the dominant effect of the central voxel in a local neighborhood (Cordes et al., 2012b; Dong et al., 2015; Friman et al., 2003; Zhuang et al., 2017; Zhuang et al., 2019). Yang, Zhuang, et al. (2018) further extend the constraints from two‐dimensional local neighborhood to three‐dimensional neighboring voxels.

### 4.3 Kernel CCA: Focusing on a nonlinear relationship between two modalities

Above CCAapplications assume joint linear relationships between two modalities; however, this assumption might not always hold in neuroscience research. Kernel CCA has been proposed to uncover the nonlinear relationship between modalities without explicitly specifying the nonlinear feature space (Equation (8)). In human research, kernel CCA has been applied to investigate the joint nonlinear relationship between simultaneously collected fMRI and EEG data (Yang, Cao, et al., 2018), to uncover gene–gene co‐association in Schizophrenia subjects (Ashad Alam et al., 2019), and to detect brain activations in response to fMRI tasks (Hardoon et al., 2007; Yang, Zhuang, et al., 2018). In preclinical research, temporal kernel CCA has been proposed to investigate the temporal‐delayed nonlinear relationship between simultaneously recorded neural (electrophysiological recording in frequency‐time space) and hemodynamic (fMRI in voxel space) signals in monkeys (Murayama et al., 2010), and to investigate a nonlinear predictive relationship between EEG signals from two different brain regions in macaques (Rodu et al., 2018) (Table 3).

**TABLE 3:** Nonlinear Kernel CCA applications

CCA variant	Modality 1	Modality 2	Reference
Kernel CCA	Brain imaging data	Brain imaging data	Yang, Cao, et al. (2018)
	Brain imaging data	Task design	Hardoon, Mourão‐Miranda, Brammer, and Shawe‐Taylor (2007); Yang, Zhuang, et al. (2018)
	Genetic information	Genetic information	Ashad Alam, Komori, Deng, Calhoun, and Wang (2019)
Temporal kernel CCA	Simultaneously recorded multiple modalities		John et al. (2017); Murayama et al. (2010); Rodu, Klein, Brincat, Miller, and Kass (2018)

Abbreviation: CCA, canonical correlation analysis.

### 4.4 Multiset CCA: More than two modalities

Multiset CCA has been specifically proposed to find common multivariate patterns across *K* modalities, with *K* > 2. The widest application of multiset CCA in neuroscience research is to uncover *covariated* patterns among demographics, clinical characteristics, behavioral measurements and multiple brain activities, including structural MRI derived measurements (gray matter, white matter, and cerebrospinal fluid densities), diffusion weighted MRI derived measurements (myelin water fraction and white matter tracts), fMRI derived measurements (static and dynamic functional connectivity, task fMRI activations, amplitude of low frequency contributions) and PET derived measurements (standardized uptake values) (Baumeister et al., 2019; Langers et al., 2014; Lerman‐Sinkoff et al., 2017; Lerman‐Sinkoff et al., 2019; Lin, Vavasour, et al., 2018; Lottman et al., 2018; Stout et al., 2018; Sui et al., 2013; Sui et al., 2015) (Table 4).

**TABLE 4:** Multiset CCA applications

CCA variant		Detailed modalities	Reference
Multiset CCA	Combine multiple brain imaging data	rsfMRI + task fMRI + sMRI	Lerman‐Sinkoff et al. (2017); Lerman‐Sinkoff, Kandala, Calhoun, Barch, and Mamah (2019)
		sMRI (WM + GM + CSF) + rsfMRI	Lottman et al. (2018)
		sMRI + fMRI + dMRI	Sui et al. (2013, 2015)
		Multiple task fMRI	Langers, Krumbholz, Bowtell, and Hall (2014)
		sMRI + fMRI + EEG	Correa, Adali, Li, and Calhoun (2010)
	Combine brain imaging data and other information	Brain imaging data (sMRI/fMRI) + neuropsychological measurements + clinical/behavioral measurements	Baumeister et al. (2019); Lin, Cocchi, et al. (2018); Lin, Vavasour, et al. (2018)
		Brain imaging data (PET + sMRI + fMRI) + neuropsychological measurements	Stout et al. (2018)
	Combine multiple subjects within a single modality	Sub1 + Sub2 + … + SubN within a single modality	Afshin‐Pour, Hossein‐Zadeh, Strother, and Soltanian‐Zadeh (2012); Afshin‐Pour, Grady, and Strother (2014); Correa, Adali, et al. (2010); Gaebler et al. (2014); Koskinen and Seppa (2014); Lankinen, Saari, Hari, and Koskinen (2014); Lankinen et al. (2016, 2018); Liu and Ayaz (2018); Varoquaux et al. (2010); Zhang, Borst, Kass, and Anderson (2017)
	Combine multiple subjects from two modalities	Sub1 + Sub2+ … + SubN from fMRI and EEG	Correa, Eichele, Adali, Li, and Calhoun (2010)
	Combine multiple ROIs within a single modality	ROI1 + ROI2 + … + ROIN within a single modality	Deleus et al. (2011)
Constraints in multiset CCA	Sparse multiset CCA	Brain imaging data + genetic information + clinical measurements	Hu, Lin, Calhoun, and Wang (2016); Hu et al. (2018); Yu et al. (2015)
	Multiset CCA with reference	Brain imaging data (fMRI + sMRI + dMRI) with neuropsychological measurements as reference	Qi et al. (2020), Qi, Calhoun, et al. (2018); Sui et al. (2018)
		Brain imaging data (fMRI + sMRI + dMRI) with genetic information as reference	Qi, Yang, et al. (2018)

Abbreviations: CCA, canonical correlation analysis; CSF, cerebrospinal fluid; dMRI, diffusion‐weighted MRI; EEG, electroencephalogram; GM, gray matter; MRI, magnetic resonance imaging; PET, position emission tomography; ROI, regions of interest; rsfMRI, resting‐state functional MRI; sMRI, structural MRI; Sub, subject; WM, white matter.

Multiset CCA has also been applied to group analysis, which combines data from multiple subjects within a single modality. In this type of applications, data from each subject are treated as one modality, and multiset CCA is used to uncover common patterns in fMRI data (Afshin‐Pour et al., 2012; Afshin‐Pour et al., 2014; Correa, Adali, et al., 2010; Varoquaux et al., 2010), consistent signals in electrophysiological recordings (Koskinen & Seppa, 2014; Lankinen et al., 2014; Lankinen et al., 2016; Lankinen et al., 2018; Zhang et al., 2017), covaried components in fNIRS data (Liu & Ayaz, 2018), and correlated fMRI and EEG signals (Correa, Eichele, et al., 2010) across multiple subjects.

Sparse multiset CCA has been applied to combine more than two variables and remove noninformative features simultaneously. Specifically, sparse multiset CCA has been applied to combine multiple brain imaging modalities with genetic information (Hao et al., 2017; Hu et al., 2016; Hu et al., 2018).

Multiset CCA *with reference* is specifically proposed as a supervised multimodal fusion technique in neuroscience research. Using neuropsychological measurements such as working memory or cognitive measurements as the reference, studies have uncovered stable covariated patterns among fractional amplitude of low frequency contribution maps derived from resting‐state fMRI, gray matter volumes derived from structural MRI and fractional anisotropy maps derived from diffusion‐weighted MRI that are linked with and can predict core cognitive deficits in schizophrenia (Qi, Calhoun, et al., 2018; Sui et al., 2018). Using genetic information as a prior reference, multiset CCA with reference has also uncovered multimodal covariated MRI biomarkers that are associated with microRNA132 in medication‐naïve major depressive patients (Qi, Yang, et al., 2018). Furthermore, with clinical depression rating score as guidance, Qi et al. (2020) have demonstrated that the electroconvulsive therapy Hdepressive disorder patients produces a covariated remodeling in brain structural and functional images, which is unique to an antidepressant symptom response. As a supervised technique, multiset CCA can be applied to uncover covariated patterns across multiple variables of special interest.

### 4.5 Other applications

CCA has also been applied in a supervised and hierarchical fashion. Zhao et al. (2017) have performed supervised local CCA with gradually varying neighborhood sizes in early autism diagnosis, and in each iteration, CCA is used to combine canonical variates from the previous step (Table 5).

**TABLE 5:** Other CCA applications

CCA variant	CCA application	Reference
Supervised local CCA	Combine two modalities	Zhao, Qiao, Shi, Yap, and Shen (2017)
Tensor CCA	Morphological networks	Graa and Rekik (2019)
Bayesian CCA	Realign fMRI data from multiple subjects	Smirnov et al. (2017)
	Task fMRI activation detection	Fujiwara, Miyawaki, and Kamitani (2013)
Others	Toolbox	Bilenko and Gallant (2016)
	Reviews	Liu and Calhoun (2014) and Sui, Adali, Yu, Chen, and Calhoun (2012)

Abbreviations: CCA, canonical correlation analysis; fMRI, functional magnetic resonance imaging.

Bayesian CCA has been used to realign fMRI activation data between actors and observers during simple motor tasks to investigate whether seeing and performing an action activates similar brain areas (Smirnov et al., 2017). The Bayesian CCA assigns brain activations to one of three types (actor‐specific, observer‐specific and shared) via a group‐wise sparse ARD prior. Furthermore, using Bayesian CCA, Fujiwara et al. (2013) establish mappings between the stimulus and the brain by automatically extracting modules from measured fMRI data, which can be used to generate effective prediction models for encoding and decoding.

More recently, in network neuroscience, Graa and Rekik (2019) propose a multiview learning‐based data proliferator that enables the classification of imbalanced multiview representations. In their proposed approach, tensor‐CCA is used to align all original and proliferated views into a shared subspace for the target classification.

## 5 ADVANTAGES AND LIMITATIONS OF EACH CCA TECHNIQUE IN NEUROSCIENCE APPLICATIONS

Table 6 explains the advantages and limitations of each CCA and its variant techniques.

**TABLE 6:** Advantages and limitations of each CCA‐related technique

Category	CCA variant	Advantages		Limitations
CCA	CCA	1) Has closed‐form analytical solution 2) Easy to apply 3) Invariant to scaling		1) Requires *N* ≫ *p*_*k*_, *k* = 1, 2 2) Signs of canonical correlations are indeterminate
Constrained CCA	Sparse CCA	1) Removes noninformative features and solves *N* ≪ *p*_*k*_ 2) Performs reasonably with high‐dimensional‐co‐linear data		Requires optimization expertise
	Structure sparse CCA	Removes noninformative features, solving *N* ≪ *p*_*k*_ with prior information about the data	1) Improves effectiveness of sparse CCA. 2) Produces biological meaningful results	1) Requires optimization expertise 2) Requires prior knowledge about the data
	Discriminant sparse CCA		Discovers group discriminant features	
	Generalized constrained CCA	1) Reduces false positives 2) Maintains most of the variance in a stable model		1) Requires optimization expertise 2) Requires predefined constraints
Nonlinear CCA	Kernel CCA	1) Finds nonlinear relationship among modalities 2) Has analytical solution		1) Requires predefined kernel functions 2) Difficult to project from kernel space back to original feature space, leading to difficulties in interpretation 3) Only linear kernel space can be projected back to the original feature space.
	Temporal kernel CCA	Most appropriate to simultaneously collect data from two modalities with time delay		
	Deep CCA	1) Finds unknown nonlinear relationship 2) Purely data‐driven		1) Requires deep learning expertise 2) Requires large number of training samples (in tens of thousands)
Multiset CCA	Multiset CCA	1) Good for more than two modalities 2) Good for group analysis		1) Requires predefined objective functions 2) The number of final canonical components does not represent the intersected common patterns across all modalities
	Sparse multiset CCA	1) Good for more than two modalities 2) Removes noninformative features and solves *N* ≪ *p*_*k*_		
	Multiset CCA with reference	Supervised fusion technique to link common patterns with a prior known variable		

Abbreviation: CCA, Canonical correlation analysis.

### 5.1 Canonical correlation analysis

#### 5.1.1 Advantages

CCA can be applied easily to two variables and solved efficiently in closed‐form using algebraic methods (Equation (3)). In CCA, the intermodality relationship is assumed to be linear and both modalities are exchangeable and treated equally. Canonical correlations are invariant to linear transforms of features in ***Y***_1_ or ***Y***_2_. In neuroscience research, CCA uncovers the joint multivariate linear relationship between two modalities and has proven to be an effective multivariate and data‐driven analysis method.

#### 5.1.2 Limitations

CCA assumes and uncovers only a linear intermodality relationship, which might not hold for neuroscience data. Furthermore, directly applying CCA requires sufficient observation support of the variables (detailed in Section 3.1). For neuroscience data, especially voxel‐wise brain imaging data, it is usually difficult to have more observations (e.g., subjects) than features (e.g., voxels). In this case, any feature in ***Y***_1_ and ***Y***_2_ can be picked up and learned by the CCA process, and directly applying CCA will produce overfitted and unstable results. ROI‐based analysis, data reduction (e.g., PCA), and feature selection (e.g., LASSO) steps are commonly applied to reduce the number of features in neuroscience data prior to CCA.

Another limitation of CCA in general is that signs of the canonical correlations and canonical coefficients are indeterminate. Solving the eigenvalue problem in Equation (3) will always give a positive canonical correlation value, and reversing the signs of ***u***_1_ and ***u***_2_ simultaneously will lead to the same canonical correlation value. Therefore, with CCA, we can only conclude that two modalities are linearly and multivariately correlated without determining the direction of the linear relationship.

### 5.2 Constrained CCA

#### 5.2.1 Advantages

Incorporating constraints in CCA can in general avoid overfitted and unstable results in CCA. More specifically, different constraints can benefit neuroscieence research in various ways.

Sparse CCA incorporates the *L*_1_‐norm penalty on the canonical coefficients ***u***_*k*_, *k* = 1, 2 such that noninformative features are automatically removed by suppressing their weights. Thus, sparse CCA is suitable for high‐dimensional co‐linear data, such as whole‐brain voxel‐wise activities or genetic data. In practice, the within‐modality covariance matrices **∑**_*kk*_, *k* = 1, 2 are replaced with the identity matrix ***I*** in sparse CCA, since estimating **∑**_*kk*_ from the high‐dimensional collinear data are both memory and time consuming. This replacement saves both computation time and physical resources, and is widely adopted in the neuroscience field.

Structure and discriminant sparse CCA removes noninformative features and incorporates prior information about the data in the algorithms simultaneously. Prior knowledge about feature structure or group assignment of each observation are required, respectively, for these two techniques. In neuroscience applications, information implanted in features can improve the performance and effectiveness of sparse CCA (Du, Liu, Zhang, et al., 2017) and guide the algorithm to produce more biologically meaningful results (Du, Huang, et al., 2016a; Liu et al., 2017). Alternatively, with group assignments implanted in each observation, discriminant sparse CCA is able to discover group discriminant features, which can later improve the performance of supervised classification (Wang et al., 2019).

Other constraints are also beneficial in neuroscience research. For instance, the *L*_2_‐norm penalty on canonical coefficients retains all features in the model with regularized weights, and therefore most of the variance can be maintained in a stable model (Dashtestani et al., 2019). In addition, when applied to task fMRI activation detection, locally constrained CCA penalizes weights on the neighboring voxels to guarantee the dominance of the central voxel and therefore, is able to reduce false positives (Cordes et al., 2012b; Zhuang et al., 2017).

#### 5.2.2 Limitations

One major limitation of constrained CCA is the requirement of expertise in optimization techniques. By having additional penalty terms on canonical coefficients or covariance matrices, analytical solutions of constrained CCA no longer exist, and, instead, iterative optimization methods are required to solve the constrained CCA problems efficiently.

The predefined constraint itself also requires prior knowledge about the data. For structure and discriminant sparse CCA, prior information about the observation domain or the feature domain is required. Furthermore, in neuroscience application, the constraint itself is usually data specific. For instance, when applying local constrained CCA to task fMRI activation detection, the predefined constraint should be strong enough to penalize neighboring voxels, but loose enough to guarantee the multivariate contribution of neighboring voxels to the central voxel. This constraint can only be selected through simulating a series of synthetic data that mimic real fMRI signals, which requires prior knowledge of the data and is time‐consuming.

### 5.3 Nonlinear CCA

#### 5.3.1 Advantages

By definition, nonlinear CCA is able to uncover multivariate nonlinear relationships between two modalities, which commonly exist in neuroscience variables. For instance, during an fMRI task, collected fMRI signals are nonlinearly correlated with the task design due to the unknown hemodynamic response function; and kernel CCA can extract this multivariate nonlinear relationship and produce a localized brain activation map (Hardoon et al., 2007).

In general, kernel CCA first implicitly transforms the original feature space into a kernel space with a predefined kernel function. With this transform, nonlinear relationship between two modalities can be discovered. Furthermore, in the new kernel space, kernel CCA can be solved efficiently with a closed‐form analytical solution.

Temporal kernel CCA shares similar advantages with kernel CCA, with additional benefits from considering temporal delays between modalities when applied to simultaneously collected data. In neuroscience research, simultaneously collected EEG/fMRI data are a typical candidate for temporal kernel CCA, as neural activities collected by fMRI data, which are the blood oxygenated level‐dependent signals, contain temporal delays caused by the hemodynamic response function (Ogawa, Lee, Kay, & Tank, 1990), as compared to the simultaneously collected EEG signals.

Deep CCA, a purely data‐driven technique, can reveal unknown nonlinear relationships between variables without assuming any predefined nonlinear intermodality relationship. It has the potential to be applied to neuroscience data that contains enough samples for training a deep learning schema.

#### 5.3.2 Limitations

For kernel CCA, a predefined kernel function needs to be selected and this selection will affect final results. This choice of kernel functions requires additional knowledge about data and the kernel function. Another major limitation of both kernel CCA and temporal kernel CCA is that it is difficult to project the kernel space (***H***_1_ and ***H***_2_) back to the original feature space (***Y***_1_ and ***Y***_2_), leading to additional difficulties in interpreting results (Hardoon et al., 2007). For instance, when applying kernel CCA to link fMRI task stimuli and collected BOLD signals for activation detection, the obtained high‐dimensional features cannot be mapped backwards to an individual voxel in order to assign the activation value because the feature embedded for commonly used nonlinear kernels (e.g., Gaussian kernel and power kernel) have information from multiple voxels. Therefore, kernel CCA with a general nonlinear kernel remains unsolved for fMRI activation analysis, and only linear kernels were used for constructing activation maps in fMRI.

Unlike kernel CCA, deep CCA does not require a predefined function and learns the nonlinear feature mapping from the data itself. However, in deep CCA, the number of unknown parameters significantly increases with the number of layers, which requires much more samples in the training data. In neuroscience data, it is usually difficult to have enough number of subjects as training samples for deep CCA. Furthermore, deep learning expertise is also required for selecting the appropriate deep learning structures for nonlinear feature mapping.

### 5.4 Multiset CCA

#### 5.4.1 Advantages

In neuroscience research, more than two variables are commonly collected for the same set of subjects. Multiset CCA uncovers multivariate joint relationships among multiple variables, which is well defined to link all collected data in this case. Furthermore, if data from one subject are treated as one modality (or variable), multiset CCA will also discover the common patterns across subjects, which becomes a powerful data‐driven group analysis method.

Sparse multiset CCA combines more than two modalities and suppresses noninformative features simultaneously, and therefore shares the advantages and limitations with both multiset CCA and sparse CCA.

Multiset CCA with reference is the only supervised CCA technique and is proposed specifically for neuroscience applications. It discovers joint multivariate relationships among variables in response to a specific reference variable. For instance, using this method, common brain changes from structural, fMRI and diffusion MRI with respect to a specific neuropsychological measurement can be discovered.

#### 5.4.2 Limitations

There are five possible objective functions for multiset CCA optimization, and different objective functions will lead to various results. The closed‐form analytical solution only exists for SUMCOR and SSQCOR objective functions. Optimization expertise are required to solve multiset CCA with other objective functions, and with constraints as well. Another major limitation of multiset CCA is that the number of final canonical components output from the algorithm does not represent the intersected common patterns across all modalities, or subjects. Instead, multiset CCA discovers the unified similarities among every modality pair (Levin‐Schwartz, Song, Schreier, Calhoun, & Adali, 2016).

### 5.5 Abstract

To summarize, conventional CCA uncovers joint multivariate linear relationships between two modalities and can be quickly and easily applied. In neuroscience research, due to the existing multiple modalities and nonlinear intermodality relationships, multiset CCA and nonlinear CCA have their own advantages when applied accordingly to appropriate variables. Constraints can be applied in these three methods to stabilize results, remove noninformative features, and produce supervised meaningful results. However, optimization expertise and prior knowledge about the data are required to select the appropriate constraints.

## 6 CHOOSING THE APPROPRIATE CCA TECHNIQUE

The first step in selecting a CCA technique is to decide what type of neuroscience application is of interest. Based on the types of combined modalities, CCA applications can be summarized into four categories (a–d): (a) finding relationship among multiple measurements; (b) detecting brain activations in response to task stimuli; (c) uncovering common patterns among multiple subjects; and (d) denoising the raw data. Table 7 summarizes current and potential techniques that can be applied for each application.

**TABLE 7:** Current applied and potential CCA techniques for each application

Applications	Currently applied	Potential techniques
Link two modalities	CCA Sparse CCA Structure/discriminant sparse CCA Kernel CCA Temporal kernel CCA	Deep CCA
Detect task fMRI activation	CCA Constrained CCA Kernel CCA	Deep CCA Sparse CCA
Uncover common patterns across multiple modalities	Multiset CCA Sparse multiset CCA	Multiset constrained CCA Deep CCA
Denoise raw data	CCA	Constrained CCA Kernel CCA Deep CCA

Abbreviations: CCA, canonical correlation analysis; fMRI, functional magnetic resonance imaging.

**FIGURE 4:**
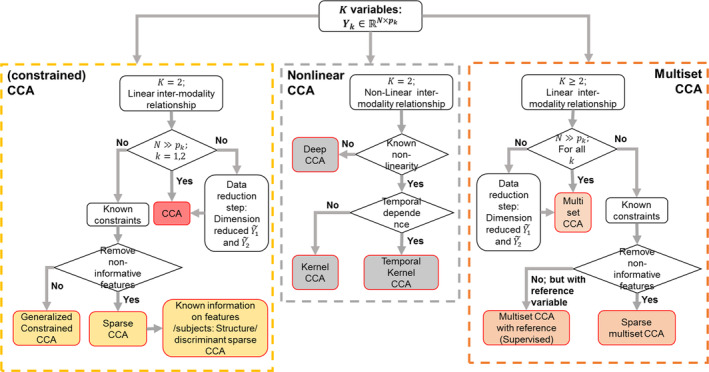
Selecting a canonical correlation analysis (CCA)‐technique that suits your application. Three scenarios are most commonly encountered in neuroscience applications: CCA with and without constraints (dashed yellow box); nonlinear CCA (dashed gray box) and multiset CCA (dashed orange box)

After determining the application of interest, the flowchart in Figure 4 provides a detailed guidance in selecting an appropriate CCA technique. Based on the number of variables (*K*) and linear or nonlinear intermodality relationships, three major applications are mostly common in neuroscience research: uncover linear relationship between two variables (dashed yellow box); find nonlinear relationship between two variables (dashed gray box) and discover covariated patterns among more than two variables (dashed orange box). Detailed choices are further made based on the number of observations and number of features within each variable, known prior knowledge about the variable, such as feature structures, and specific questions of interest for research studies.

Furthermore, here, we give an experimental example of CCA applications in neuroscience research.

Among many neuroscience applications, CCA is commonly used as a data fusion technique to uncover the association between two datasets. In the following, we demonstrate how to follow the guidance in Figure 4 to link disease‐related pathology using fMRI and structural MRI data from cognitive normal subjects and subjects with mild cognitive impairment (MCI). As a prodromal stage of Alzheimer's disease, both functional and structural pathology are expected in MCI subjects. Yang, Zhuang, Bird, et al. (2019) used CCA to examine the disease‐related links between voxel‐wise functional information (e.g., eigenvector centrality mapping from fMRI data, $X_{1}\in R^{N\times p_{1}}$) and voxel‐wise structural information (e.g., voxel‐based morphometry from T1 structural MRI data, $X_{2}\in R^{N\times p_{2}}$), where *N* is the number of subjects, and *p*_1_ and *p*_2_ are the number of voxel‐wise features for fMRI and structural MRI data, respectively. Since there are only two imaging modalities in the analysis, multiset CCA is not an option for this case. Considering that deep CCA requires a large number of samples but *N* ≪ *p*_1_ or *p*_2_, and kernel CCA has the difficulty to project coefficients back to original voxel‐wise feature space as mentioned in Section 5.3, a linear relationship between these two imaging modalities is considered. There are two approaches for the scenario that the number of samples is much less than the number of features.

**FIGURE 5:**
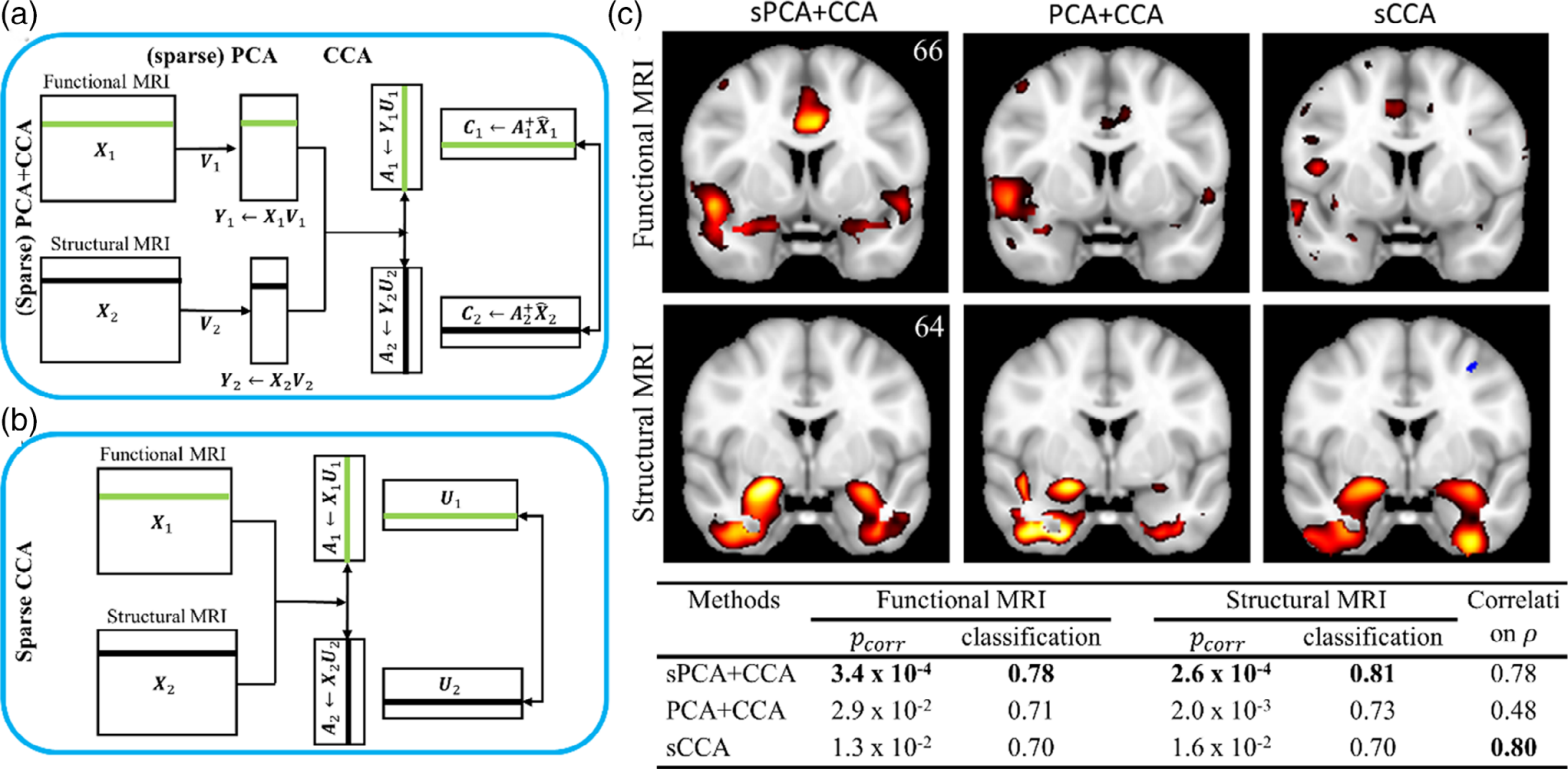
Example of choosing canonical correlation analysis (CCA) variants by following the guideline. Voxel‐wise functional and structural MRI information from cognitive normal subjects and subjects with mild cognitive impairment were used for data fusion analysis. (a) Schematic diagram of (sparse) principal component analysis (PCA) + CCA. The abbreviation sPCA stands for sparse PCA. (b) Schematic diagram of sparse CCA (sCCA). (c) Top panel shows the most disease‐discriminant functional and structural component and the bottom panel shows the correlation between datasets (*ρ*), the significance of the correlation derived from nonparametric permutation test (*p*_corr_) and the classification accuracy for each method

The first approach is to perform dimension reduction before feeding data into conventional CCA as shown in Figure 5a. Yang, Zhuang, Bird, et al. (2019) used PCA or sPCA (Witten et al., 2009) for dimension reduction and then fed CCA with dimension‐reduced data ***Y***_1_ and ***Y***_2_. CCA found a set of canonical coefficients ***U***_*k*_, *k* = 1, 2 and the corresponding canonical variables ***A***_*k*_. The voxel‐wise weight coefficient can be obtained with a pseudo inverse operation. The other approach is to implement constrained CCA as shown in Figure 5b. With the assumption that a proportion of voxels in the brain is not informative for finding the association between fMRI and structural MRI data, sparse CCA was applied with ***X***_1_ and ***X***_2_ directly without dimension reduction step (Yang, Zhuang, Bird, et al., 2019). The canonical coefficients ***U***_*k*_, *k* = 1, 2 are in the voxel‐wise feature space, thus no operation is required to calculate voxel‐wise weight coefficients.

The voxel‐wise weight coefficients play a role in uncovering which brain regions are most relevant for finding the association between datasets. The voxel‐wise weight maps for the most significant disease‐related component in ***A***_*k*_ for (s)PCA + CCA and sparse CCA is shown in Figure 5c. A nonparametric permutation test is applied to test the significance of the association between fMRI and structural MRI data with *p* values shown at the bottom of Figure 5c. In this study, the canonical variables ***A***_*k*_ computed from sPCA + CCA have the highest classification accuracy for both fMRI and structural MRI data.

## 7 FUTURE DIRECTION OF CCA IN NEUROSCIENCE APPLICATIONS

Currently, when applying CCA to data with a smaller number of observations than features, either a data reduction orfeature selection step is performed as a preprocessing step, or an *L*_1_ norm penalty is added as a constraint to remove noninformative features. Future efforts should be made toward incorporating prior information on feature structures of input variables that are more reasonable or more biological meaningful, and canonical correlation values should be computed in a one step process that includes prior information. Furthermore, applying CCA and its variant techniques to uncover joint multivariate relationships between two modalities has dominated the current CCA applications in the neuroscience field. In these applications, various techniques have been proposed to incorporate prior information within variables to boost the model performance, such as considering group‐discriminant features to strengthen group separation. However, much less effort was put to incorporate these prior information within the variables in multiset CCA. In neuroscience research, collecting multiple modalities of a single subject has become a commonplace, and with more than two variables, multiset CCA should be considered for this multimodal data‐fusion more often. Future efforts toward incorporating prior information within each variable to further improve the performance of multiset CCA could shed new lights in neuroscience research. For instance, we suggest incorporating group information in multiset CCA to extract common group‐discriminant patterns among multiple measurements derived from fMRI, or to uncover correlated group‐discriminant feature among brain imaging data and behavioral or clinical measurements. Furthermore, nonlinear relationships among multiple modalities have not been explored within multiset CCA in neuroscience research. It might be of interest to incorporate kernels in multiset CCA to uncover covariated nonlinear patterns among multiple brain imaging data, or to input each variable through multiple layers to generate “deep” features before applying multiset CCA.

In addition, future efforts are also required to statistically interpret CCA results. Currently, a parametric statistical significance of CCA model is only well defined for conventional CCA. Statistical significances of CCA variants are usually determined nonparametrically through permutation tests, which are time‐consuming and methods dependent. Furthermore, even using permutation tests, statistical significance can only be determined for each canonical correlation value, instead of canonical coefficients. Therefore, we cannot determine the statistical significance of a specific feature in the model. Identifying important features as potential biomarkers is usually an end goal in neuroscience. Therefore, developing test statistics to interpret CCA results by determining statistically important features would also benefit neuroscience research.

## 8 CONCLUSION

Uncovering multivariate relationships between modalities of the same subjects have gained significant attentions in neuroscience research. CCA is a powerful tool to investigate these joint associations and has been widely applied. Multiple CCA‐variant techniques have been proposed to fulfill specific analysis requirements. In this study, we reviewed CCA and its variant techniques from a technical perspective, with summarized applications in neuroscience research. Of each CCA‐related technique, detailed formulation and solution, relationship with other techniques, current applications, advantages, and limitations are provided. Selecting the most appropriate CCA‐related technique to take full advantages of available information embedded in every variable in joint multimodal research might shed new lights in our understandings of normal development, aging, and disease processes.

## 9 CODE AVAILABILITY

Python‐based CCA toolbox (Bilenko & Gallant, 2016) is available on github: http://github.com/gallantlab/pyrcca; CCA package in R can be found in González, Déjean, Martin, and Baccini (2008). Codes for applying CCA and kernel CCA to detect task‐fMRI activations are available on github (Yang, Zhuang, et al., 2018; Zhuang et al., 2017): https://github.com/pipiyang/CCA_GUI. Bayesian CCA with group‐wise ARD prior and the relevant techniques are implemented in R CCAGFA package (https://cran.r-project.org/web/packages/CCAGFA/index.html).

## Data Availability

There is no data or code involved in this review article.
